# Interface‐Adaptive Dual‐Color Hydrogel with Self‐Repairing Function and High Adhesion as Flexible Wearable Sensor for Minimally‐Invasive Monitoring Pesticide Residue in Living Crop

**DOI:** 10.1002/advs.202512386

**Published:** 2025-10-13

**Authors:** Jianling Chen, Zihan Zhu, Yafei Li, Zizhe Wang, Wendong Wang, Hong Chen, Xuelian Xin, Zhenguo Chi, Haiyin Li

**Affiliations:** ^1^ Hebei Key Laboratory of Public Health Safety School of Public Health Hebei University Baoding Hebei 071002 China; ^2^ State Key Laboratory of New Pharmaceutical Preparations and Excipients Key Laboratory of Medicinal Chemistry and Molecular Diagnosis of the Ministry of Education College of Chemistry and Materials Science Hebei University Baoding Hebei 071002 China; ^3^ Luoyang Key Laboratory of Organic Functional Molecules College of Food and Drug Luoyang Normal University Luoyang Henan 471934 China; ^4^ School of Environmental and Chemical Engineering Wuyi University Jiangmen Guangdong 529020 China

**Keywords:** dual‐color hydrogel, high adhesion, interface self‐adaptability, self‐repairing function, wearable crop sensor

## Abstract

Harnessing dandy mechanical property, hydrogels facilitate the construction of wearable fluorescence sensors for minimally‐invasive monitoring pesticide residue in living crops, while the interface self‐adaptability and detection accuracy remain hugely challenging. Herein, blue‐emission aggregation‐induced emission nanoparticles (*b*‐TPE NPs) and red‐emission Mn‐doped ZnS quantum dots (*r*‐Mn@ZnS QDs) are encapsulated inside agarose, borax, and polyvinyl alcohol‐co‐constituted hydrogel to construct dual‐color TPE@Mn@ZnS@AG@PVA as wearable crop sensor. Owing to specific recognition of *r*‐Mn@ZnS QDs by thiophannate‐methyl (TM), the customized TPE@Mn@ZnS@AG@PVA shows a gradual color evolution from reddish purple to blue with high resistance to environmental and experimental interferences via quenching *r*‐Mn@ZnS QDs’ fluorescence by target‐induced aggregation and photo‐induced electron transfer while acting negligible disturbance in blue fluorescence of *b*‐TPE NPs, consequently achieving TM dual‐color assay with limit of detection at 0.045 µg mL^−1^. Additionally, TPE@Mn@ZnS@AG@PVA fascinates smart interface self‐adaptability, high adhesion, and outstanding self‐repairing function, and then is pasted onto the interfaces of crops to deliver on the sense pesticide residue data in minimally‐invasive manner, leading to the monitor of dynamic TM degradation. This study offers an in‐depth penetration into dual‐color wearable sensor with distinctive features for minimally‐invasive monitoring pesticide residue in living crops, advancing the development of wearable crop sensors and precision agriculture.

## Graphical abstract

Dual‐color hydrogel TPE@Mn@ZnS@AG@PVA is prepared using *b*‐TPE NPs and *r*‐Mn@ZnS QDs as blue‐channel and red‐channel indicators, exhibits smart interface adaptability, high adhesion, good self‐repairing function, and superior response to thiophannate‐methyl, and thus acts as a flexible wearable sensor for in situ, on‐site and minimally‐destructive detection of pesticide residue in living crop via picturing the images and intelligent signal processing.

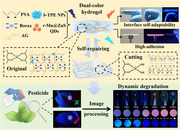

## 1 Introduction

Agrochemistry revolution in the past decades tremendously promotes the agricultural advancement of increasing yield/revenue and relieving food scarcity.^[^ ^1^ ^]^ Unfortunately, excessive use or abuse begets the residue of these agrochemicals in farm plant, environmental water, and soil, consequently resulting in serious burden to food safety and human health.^[^ ^2^ ^]^ For example, pesticides are widely used for preventing the damage of disease, pest and grass on agricultural crops,^[^ ^3^ ^]^ but over reliance makes people expose to pesticides residues, thereby inducing the occurrence and deterioration of diseases associated with respiratory, digestive, urinary and nervous systems, even cancers and death.^[^ ^4^, ^5^, ^6^, ^7^ ^]^ These findings highlight the crucial request to pursue state‐of‐the‐art protocols for quantitatively monitoring pesticide residue concentration in agricultural crops,^[^ ^8^ ^]^ which interprets a feeling of how to perfect the resource investment and management while maximizing food safety as well as crop yield and quality.^[^ ^9^ ^]^ The available cutting‐edge sensors for pesticide residue mainly count upon the physiochemical properties of signal indicators in combination with chromatograph, UV‐vis absorption spectrometer, fluorescence spectrometer, electrochemical workstation and electrochemiluminescence spectrometer.^[^ ^10^, ^11^, ^12^ ^]^ Despite the rapid progress, these protocols are subjected to large‐scale and high‐price instruments, professional workers and tedious operation, and necessitate the test sample to be completely destructed, where the extract must be separated, purified and concentrated.^[^ ^13^, ^14^, ^15^ ^]^ Furthermore, pesticide residue concentration in living crops changes timely.^[^ ^16^ ^]^ In this regard, it is highly desirable to propose no/minimally‐invasive strategies for on‐site compassing the pesticide residue information from agricultural crops without complicated sample pre‐treatments.

Flexible wearable sensors, mainly constituted by signal indicator and hydrogel matrix, have the merits of ease operation, high adhesion, portability and tailored shape,^[^ ^17^, ^18^, ^19^ ^]^ and thus are considered ideal candidates for no/minimally‐invasive detection of various analytes from human, animal and plant.^[^ ^20^, ^21^, ^22^, ^23^, ^24^ ^]^ From this context, they open up a promising avenue to address the focused tasks laying in current pesticide residue assays:^[^ ^25^ ^]^ harnessing unique mechanical compatibility,^[^ ^26^, ^27^ ^]^ hydrogel is mounted onto the surface of leaf, stem and skin of crops,^[^ ^28^ ^]^ in which analyte diffuses inside the 3D ducts of hydrogel and comes across the signal indicators to vary readout signal, on‐site reporting pesticide residue information for quantitative detection.^[^ ^29^, ^30^ ^]^ In view of functional diversity, embedding different indicators into hydrogel may advance different flexible wearable sensors.^[^ ^31^, ^32^, ^33^, ^34^ ^]^ At present, considerable efforts have witnessed incorporating fluorescent emitters into hydrogels to construct wearable sensors is a straightforward thinking for in situ and on‐site detection of pesticide residue.^[^ ^35^ ^]^ Compared with others, wearable fluorescence sensors not only feature easy operation, high sensitivity and visualization but also can handily convert fluorescence color information into red (R), green (G) and blue (B) values in conjunction with a smart‐phone,^[^ ^36^ ^]^ resulting in substantially improved practical usability.^[^ ^37^ ^]^ Nevertheless, the bulk of wearable fluorescence sensors undergo the change in intensity of single color rather than dual colors, and so are effortlessly disturbed by experimental/environmental factors to cause false positive result.^[^ ^38^ ^]^ Furthermore, many fluorescence emitters easily agglomerate in hydrogel matrix to quench fluorescence emission because of aggregation‐caused quenching (ACQ) effect;^[^ ^39^ ^]^ pesticide residue assays usually rely on bioenzyme/antibody/aptamer/molecularly imprinted technique to achieve reliable detection,^[^ ^40^, ^41^ ^]^ and then cannot match the requirement of rapid, low‐cost and on‐site detection.^[^ ^42^ ^]^ Thus, constructing dual‐color wearable sensors for on‐site and no/minimally‐destructive detection of pesticide residue via introducing high‐property emitters to inhibit ACQ meanwhile keeping high sensitivity and good selectivity is largely urgent but remains challenging.

**Scheme 1:**
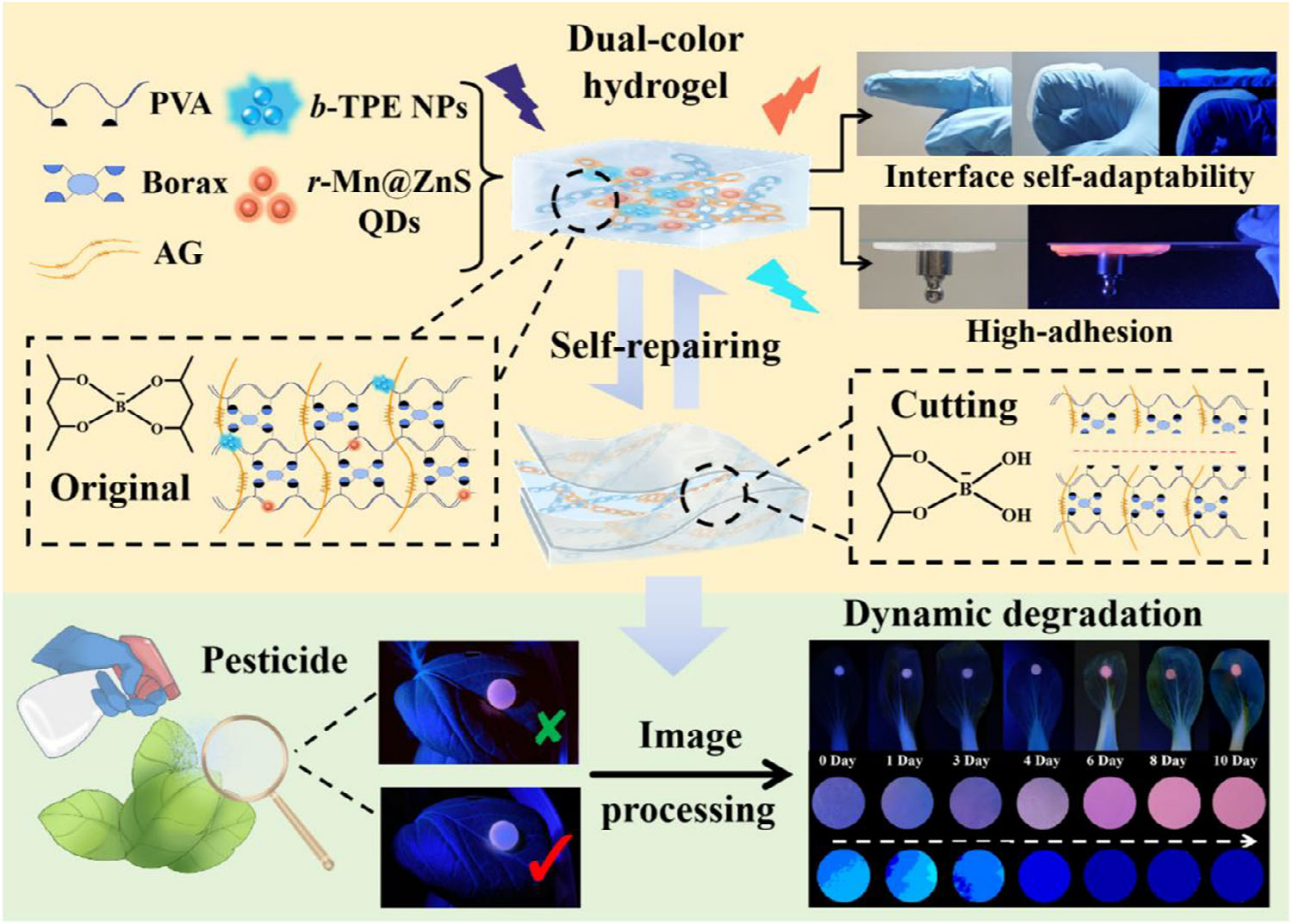
Diagram illustration of preparation of dual‐color multi‐functional hydrogel TPE@Mn@ZnS@AG@PVA for minimally‐destructive detection of pesticide residue in living crop.

Herein, we design and construct a dual‐color fluorescence hydrogel TPE@Mn@ZnS@AG@PVA with smart interface self‐adaptability, high adhesion, intelligent self‐repairing function and unique stimuli‐response property by decorating aggregation‐induced emission (AIE) nanoparticles (*b*‐TPE NPs) and Mn‐doped ZnS quantum dots (*r*‐Mn@ZnS QDs) into agarose (AG), polyvinyl alcohol (PVA) and borax‐constituted hydrogel (**Scheme** **1**). *b*‐TPE NPs and *r*‐Mn@ZnS QDs respectively showed gleaming blue and red fluorescence emission, and then are feasible to propose dual‐color system resistant to ACQ and interference of background self‐luminescence. Interestingly, the dual‐color system displayed preferable response to pesticide thiophanate‐methyl (TM), in which the red fluorescence of *r*‐Mn@ZnS QDs was quenched via aggregation and photo‐induced electron transfer (PET) accompanied with negligibly changed blue fluorescence of *b*‐TPE NPs, leading to ratiometric dual‐signal assay with improved anti‐interference ability. AG, borax and PVA‐co‐assembled double‐network hydrogel featured high transparency, multiple porosity, high water permeability, towering mechanical property, as well as outstanding self‐repairing function and interface self‐adaptability, which highlighted TPE@Mn@ZnS@AG@PVA acting as a wearable sensor for in situ, rapid and minimally‐destructive detection of TM in bok choy via picturing fluorescence color ranging from reddish purple to blue and intelligent signal processing. This work renders a new protocol to construct dual‐color wearable sensors for minimally‐invasive detection of pesticide residue in living crops and instructs how to engineer the next‐generation wearable crop devices, promoting the rapid advancement of wearable sensors in precision agriculture with prospective mileage for food protection.

## 2 Results and Discussion

### 2.1 Design and Characterization of Dual‐Color System

**Figure 1:**
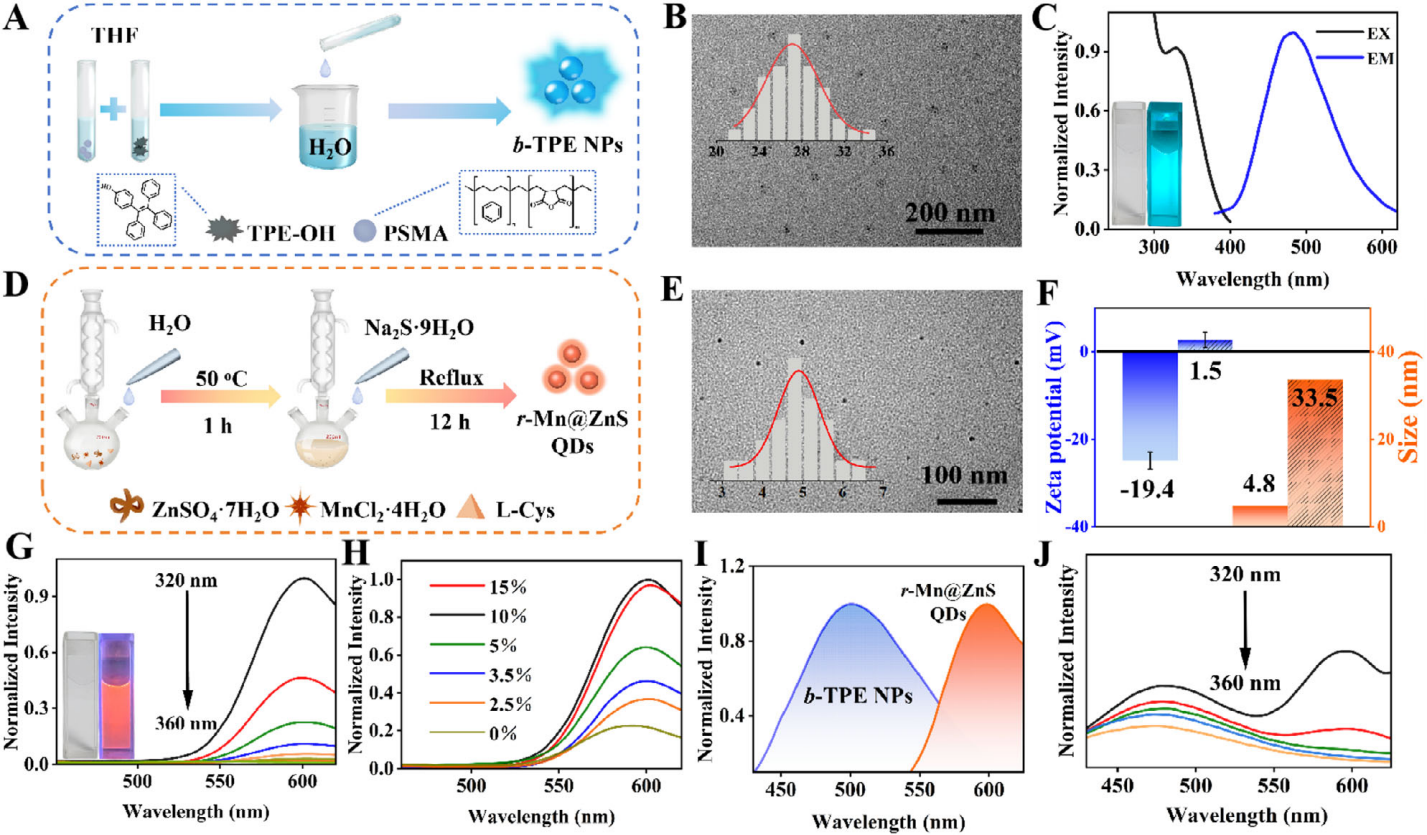
Schematic diagram of synthesis of *b*‐TPE NPs A) and *r*‐Mn@ZnS QDs D). TEM image and size distribution histogram of *b*‐TPE NPs B) and *r*‐Mn@ZnS QDs E). C) Excitation and emission spectra of *b*‐TPE NPs, and insets showed the daylight (left) and fluorescence (right) images of *b*‐TPE NPs. F) Zeta potential and size of *r*‐Mn@ZnS QDs (left) and *r*‐Mn@ZnS QDs+TM (right). G) Fluorescence curves of *r*‐Mn@ZnS QDs under the excitation of light with different wavelength, and insets showed the daylight (left) and fluorescence (right) images. H) Fluorescence curves of *r*‐Mn@ZnS QDs prepared in the presence of MnCl_2_·4H_2_O with different mass ratio of ZnSO_4_·7H_2_O. I) Normalized fluorescence spectra of *b*‐TPE NPs and *r*‐Mn@ZnS QDs. J) Fluorescence spectra of TPE NPs‐Mn@ZnS QDs under the excitation of light with different wavelength.

As is well known that people's eyes are higher sensitive to variation in fluorescence color than in strength. Aiming to construct dual‐color wearable sensors, the indicators must simultaneously exhibit two non‐interfering fluorescence signals under the same light excitation. *b*‐TPE NPs emitting fluorescence at blue channel and *r*‐Mn@ZnS QDs emitting fluorescence at red channel are chosen as signal indicators. The procedure for synthesizing *b*‐TPE NPs was illustrated in **Figure** **1**A. TPE‐OH, a typical AIE molecule (Figure S1, Supporting Information), acted as the fluorescence indicator; in stark contrast to TPE‐OH, poly(styrene‐co‐maleic anhydride) (PSMA) displayed no any fluorescence emission (Figure S2A, Supporting Information) and acted as the stabilizing agent to supply ─COOH group. Upon the addition of THF solution containing TPE‐OH and PSMA into water, hydrophobic fragments in TPE‐OH and PSMA gradually aggregated into the core with surface ─COOH, contributing to formation of nano‐structured aggregates *b*‐TPE NPs with size of ≈28 nm (Figure 1B). Optical characterizations in Figure 1C suggested *b*‐TPE NPs displayed gleaming blue fluorescence with maximum emission wavelength at 480 nm and excitation wavelength at 320 nm, which were approximately the same as that of TPE‐OH (Figure S2B, Supporting Information), and changing excitation wavelength from 310 nm to 360 nm exerted negligible disturbance on emission wavelength, but varied intensity obviously (Figure S3, Supporting Information). pH value and ion strength‐involved characterizations in Figure S4A,B (Supporting Information) showed *b*‐TPE NPs enjoyed cracking stability and anti‐interference ability according to slightly variable fluorescence intensity, then suggesting the huge potential for application in complex plant samples. According to surface zeta potential of −24.9 mV and no observed precipitate after storing for 24 h (Figure S4C, Supporting Information), *b*‐TPE NPs enjoyed fantastic water dispersion, which might be due to the presence of surface ─COOH justified by FT‐IR characterization again (Figure S5A, Supporting Information). In addition, X‐ray photoelectron spectroscopy (XPS) analysis showed *b*‐TPE NPs mainly included C and O elements, in which O 1s peak was spited into 532.0 eV (C═O) and 532.9 eV (C─O), and C 1s peak was spited into 285.0 eV (C─C), 286.3 eV (C─O) and 288.7 eV (C═O), indicating the presence of ─COOH again (Figure S6, Supporting Information) and aligning well with the compositions of raw materials.

Compared with organic/polymeric fluorescence dyes, QDs feature relatively large Stokes shift, and thus have a great potential to integrate with *b*‐TPE NPs for proposing dual‐color system. Whereas, the research of QDs mainly relies on Cd element, which exhibits high toxicity to hinder their widespread application. So with this in mind, non‐Cd QDs *r*‐Mn@ZnS QDs were synthesized via hydrothermal reaction with schematic illustration in Figure 1D. TEM morphology in Figure 1E revealed the emergence of nano‐spherical aggregates with diameter of ≈4.8 nm and zeta potential of ‐19.4 mV, implying the formation of *r*‐Mn@ZnS QDs with outstanding water dispersion. This might be taken sides with L‐Cys that was linked onto *r*‐Mn@ZnS QDs’ surface (Figure S5 B, Supporting Information). In addition, Mn, Zn, S, C, N, and O elements were recorded from XPS characterization (Figure S7, Supporting Information). Further, Mn 2p was deconvoluted into two peaks including 640.4 and 654.3 eV, indicating the presence of Mn^2+^. On the other hand, Zn 2p was fitted into 1021.3 and 1044.3 eV, and S 2p was fitted into 161.5 and 162.6 eV. Based on this, it was concluded that the produced QDs was Mn@ZnS rather than other metal‐based sulfides. Fluorescence characterizations in Figure 1G suggested *r*‐Mn@ZnS QDs emitted red luminescence with maximum emission wavelength at 595 nm with high resistance to ion strength (Figure S8, Supporting Information), and changing excitation wavelength from 320 to 360 nm only weakened fluorescence intensity. Notably, with Mn^2+^ amount increasing, the peak intensity of *r*‐Mn@ZnS QDs at 595 nm initially became higher and finally slightly varied (Figure 1H), indicating the preferable mass ratio was 10%. At the same time, high fluorescence intensity in the range of 3.0–11.0 was measured, while declining or increasing the pH value resulted in weaker fluorescence emission (Figure S9, Supporting Information), might attributable to the difference in protonation/deprotonation of *r*‐Mn@ZnS QDs induced by different pH value.

Obviously, *b*‐TPE NPs have the signal in the range of 400–600 nm with peak at 480 nm, and *r*‐Mn@ZnS QDs fluoresce ranging from 530 to 630 nm with peak at 595 nm. Thus, both signals didn't interference with each other, meeting the basic requirement of constructing dual‐color system (Figure 1I). Thus, the fluorescence emission behavior of mixed *b*‐TPE NPs and *r*‐Mn@ZnS QDs (TPE NPs‐Mn@ZnS QDs) was studied. When the excitation wavelength was 360 nm, fluorescence signal at 480 nm attributable to *b*‐TPE NPs was observable, nevertheless there was no any signal at 595 nm attributable to *r*‐Mn@ZnS QDs measured (Figure 1J). Declining the excitation wavelength to 350 and 340 nm gradually heightened the blue‐channel intensity while further keeping approximately zero red‐channel fluorescence. Only when the excitation wavelength was ≤330 nm, fluorescence emission band at 595 nm appeared, and both signal intensities of 480 and 595 nm were high under the irradiation of 320 nm light. Taking these phenomena into account, 320 nm light was preferably used to excite TPE NPs‐Mn@ZnS QDs for showing the dual‐color fluorescence emission.

### 2.2 Enquiring the Performance of Dual‐Color Sensor

**Figure 2:**
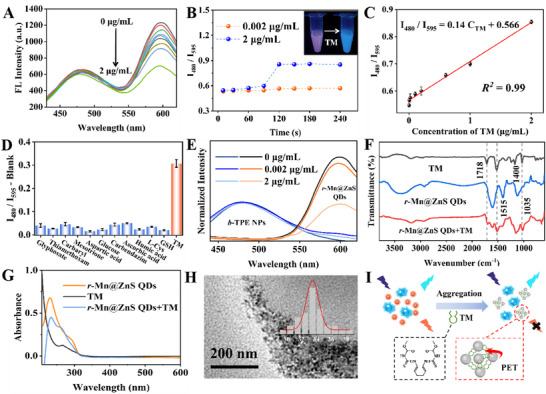
A) Fluorescence spectra of TPE NPs‐Mn@ZnS QDs exposing to TM with different concentrations. B) Dynamic response of TPE NPs‐Mn@ZnS QDs to TM and corresponding fluorescence images. C) Linear curve between I_480_/I_595_ and C_TM_. D) Selectivity investigation according to exported I_480_/I_595_‐Blank in the presence of different analytes. Blank meant the value of I_480_/I_595_ in the absence of TM. E) Fluorescence spectra of *b*‐TPE NPs or *r*‐Mn@ZnS QDs in the presence of TM. F) FT‐IR spectra of different samples. G) UV‐vis spectra of different samples. H) TEM morphology and size distribution of *r*‐Mn@ZnS QDs in the presence of TM. I) Sketch map of working principle of TPE NPs‐Mn@ZnS QDs for TM dual‐color detection.

The above unique characteristics made TPE NPs‐Mn@ZnS QDs greatly favorable to construct a dual‐color sensor for quantitatively detecting pesticide, using TM as proof‐of‐concept analyte. As manifested in **Figure** **2**A, TPE NPs‐Mn@ZnS QDs simultaneously displayed intense blue‐channel and red‐channel fluorescence signals. Upon the accession of TM, fluorescence intensity of *r*‐Mn@ZnS QDs became weaker and weaker while sustaining negligible variation in blue‐channel intensity of 480 nm. Time‐dependent characterization in Figure 2B suggested the ratio between blue channel and red channel (I_480_/I_595_) increased rapidly to finish analysis within 2 min for 0.002 and 2.0 µg mL^−1^ TM, accompanied with fluorescence color variation from reddish purple to blue (inset). Figure 2C depicted a linear relationship between I_480_/I_595_ and TM concentration (C_TM_) in the range of 0.002‐2 µg mL^−1^, with working equation of I_480_/I_595_ = 0.14 C_TM_ + 0.566 and co‐efficient of 0.99. The limit of detection (LOD) was further calculated to be 0.54 ng mL^−1^ based on 3σ rule, which was lower than those of reported sensors for recognizing TM based on single signal (Table S1, Supporting Information). Such low LOD might be originated from *r*‐Mn@ZnS QDs’ bright fluorescence emission and unique surface molecule structure, which allowed for an obvious intensity variation in the presence of only a small amount of TM. Furthermore, according to slightly changed I_480_/I_595_, glyphosate, thiamethoxam, carbaryl, mesotrione, aspartic acid, glucose, carbendazim, ascorbic acid, humic acid, L‐Cys and GSH with structures in Figure S10 (Supporting Information) didn't interfere the response of TPE NPs‐Mn@ZnS QDs to TM, implying the good selectivity (Figure 2D). The co‐existence of glyphosate/thiamethoxam/carbaryl/carbendazim/KCl/glucose/glutamic acid/humic acid/ascorbic acid/L‐Cys/GSH with TM could also not vary the exported I_480_/I_595_ value, indicating the strong anti‐interference ability (Figure S11, Supporting Information). Taken together, TPE NPs‐Mn@ZnS QDs‐based dual‐color sensor for TM fascinated high sensitivity, good selectivity and strong anti‐interference ability.

To gain deep insight into sensing principle, individual *b*‐TPE NPs or *r*‐Mn@ZnS QDs were used to detect TM. Fluorescence spectra in Figure 2E suggested TM apparently silenced the red‐channel fluorescence of *r*‐Mn@ZnS QDs, but made hardly influence in blue‐channel intensity of *b*‐TPE NPs, which was in accordance with fluorescence variation of TPE NPs‐Mn@ZnS QDs in the presence of TM, justifying the response to TM was based on *r*‐Mn@ZnS QDs rather than *b*‐TPE NPs. In this context, the interaction mechanism between *r*‐Mn@ZnS QDs and TM was thoroughly studied. As manifested in Figure 2F, typical FT‐IR peaks at 1718, 1515, and 1035 cm^−1^ attributable to C═O, benzene skeleton and C═S, emerged in TM‐treated *r*‐Mn@ZnS QDs compared to *r*‐Mn@ZnS QDs alone, indicating the effective anchor of TM onto *r*‐Mn@ZnS QDs’ surface being judged by variable zeta potential from −19.4 to 1.5 mV again (Figure 1F). Further, the addition of TM enabled the emergence of SERS signals at 1020 and 1452 cm^−1^ (Figure S12, Supporting Information), and XPS peaks of N─C═O at 400.1 eV and C═S at 164.1 eV (Figure S7, Supporting Information), and not only declined the absorbance of *r*‐Mn@ZnS QDs at 230 nm, but also led to the disappearance of shoulder peak at 300 nm (Figure 2G). All these information revealed TM was adsorbed onto the surface *r*‐Mn@ZnS QDs to produce new composites, in which PET from *r*‐Mn@ZnS QDs to TM was switched according to the energy level analysis (Figure S13, Supporting Information), consequently quenching the fluorescence emission. Besides, the morphology became irregular with raised size from 4.8 to 33 nm, implying the efficient aggregation of *r*‐Mn@ZnS QDs triggered by TM (Figure 2H). Based on the above information, the detection principle of TPE NPs‐Mn@ZnS QDs to TM was anticipated (Figure 2I): TM with four ─NH‐ group bears positive charge, and *r*‐Mn@ZnS QDs with surface ─COOH group exhibit negative charge; with TM being added, electrostatic attraction and hydrogen binding happened to induce the aggregation of *r*‐Mn@ZnS QDs leading to the variation in morphology, size and surface charge, and meanwhile PET from *r*‐Mn@ZnS QDs to TM was switched because of close contact, resulting in quenched red‐channel fluorescence accompanied with slightly disturbed blue‐channel fluorescence.

### 2.3 Performance Characterization of Dual‐Color Hydrogel

**Figure 3:**
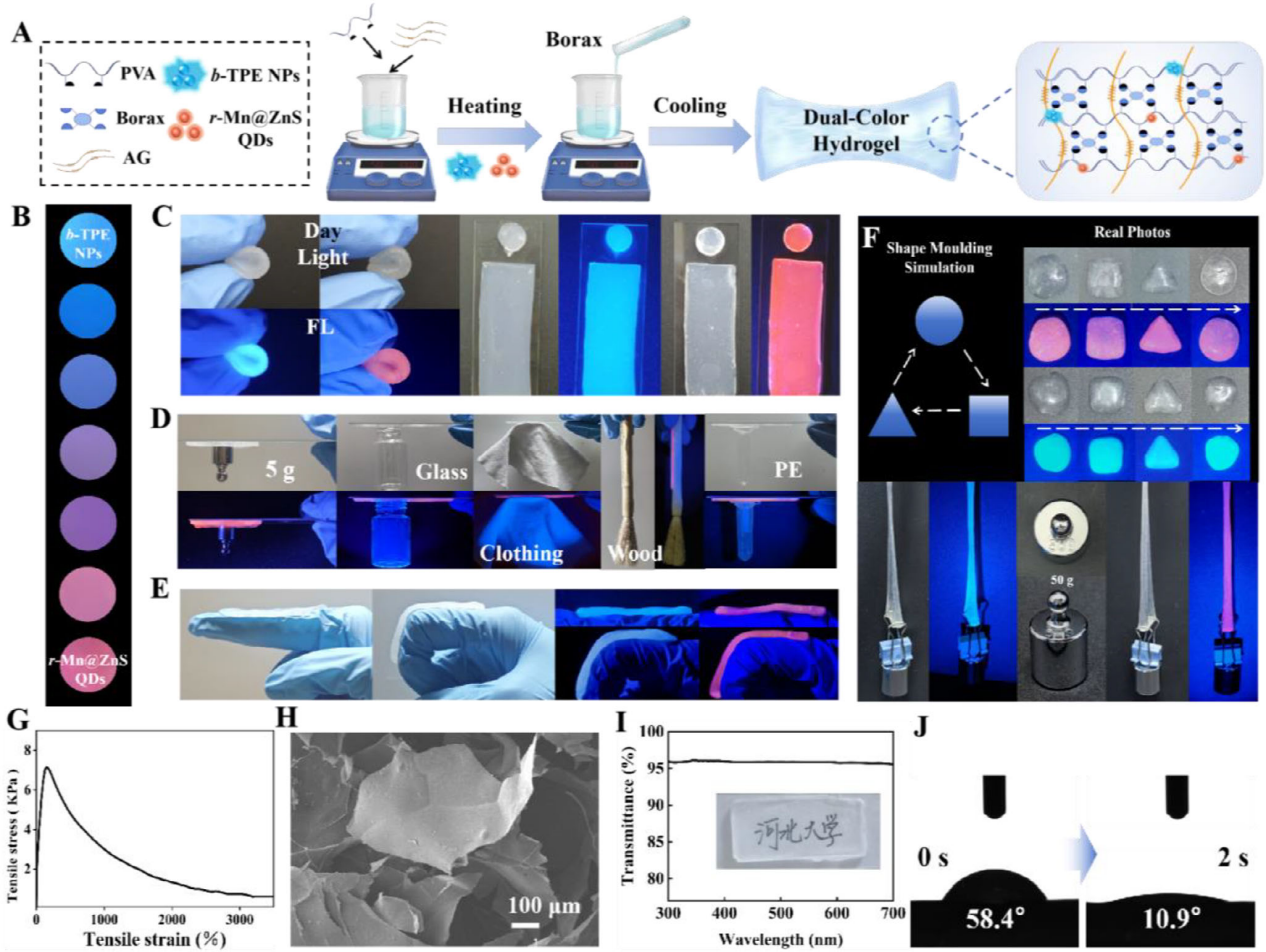
A) Diagrammatic sketch of construction of PVA, AG and boroax‐co‐constituted dual‐color hydrogel by embedding *b*‐TPE NPs and *r*‐Mn@ZnS QDs. B) Fluorescence images of hydrogel based on *b*‐TPE NPs and *r*‐Mn@ZnS QDs with different volume ratio (5:1, 2:1, 1:1, 1:2, and 1:5). C) Daylight and fluorescence images of the as‐constructed hydrogel with different shapes. D) Daylight and fluorescence images of different materials‐adhered hydrogel. E) Interface adaptability of hydrogel on finger joint. F) Daylight and fluorescence images of hydrogels being processed into different shapes and stretched by 50 g balance weight. G) Stress‐strain curve of TPE@Mn@ZnS@AG@PVA. H) SEM morphology of freeze‐dried TPE@Mn@ZnS@AG@PVA. I) Transmittance of TPE@Mn@ZnS@AG@PVA versus different wavelength. J) Water contact angel of TPE@Mn@ZnS@AG@PVA versus time.

Considering the low cost and good biocompatibility of AG and PVA, and slight disturbance on fluorescence emission of *b*‐TPE NPs and *r*‐Mn@ZnS QDs (Figure S14, Supporting Information), *b*‐TPE NPs and *r*‐Mn@ZnS QDs were decorated into matrix containing AG, PVA, and borax to construct the dual‐color hydrogel (**Figure** **3**A). Rising temperature to make AG and PVA dissolve in water, and then *b*‐TPE NPs and *r*‐Mn@ZnS QDs were added to get the uniformly dispersed solution via stirring. After that, borax was introduced to react with PVA via high‐affinity interaction between boronic acid and cis‐diol for yielding cross‐linked PVA as first network. Meanwhile, at low temperature, AG molecules reacted with each other to form hydrogel as second network. In such process, *b*‐TPE NPs and *r*‐Mn@ZnS QDs were immobilized inside the double‐network hydrogel, thus generating dual‐color hydrogel TPE@Mn@ZnS@AG@PVA. The fluorescence emission color could be readily regulated via changing the amounts of *b*‐TPE NPs and *r*‐Mn@ZnS QDs. As illustrated in Figure 3B, individual *b*‐TPE NPs‐embedded hydrogel displayed blue fluorescence color, and increasing the amounts of *b*‐TPE NPs advanced the fluorescence color strength (Figure S15A, Supporting Information); in contrast, only brilliant red fluorescence was observed for *r*‐Mn@ZnS QDs‐embedded hydrogel (Figure S15B, Supporting Information); improving the usage ratio between *r‐*Mn@ZnS QDs and *b‐*TPE NPs promoted the fluorescence color varying from blue to red with a shading process. As expected, hydrogels with different shapes were prepared in specific molds, and could be bent, compressed, and stretched without any disturbance on fluorescence color (Figure S16, Supporting Information), indicating the good flexibility and processing ability (Figure 3C). Furthermore, such hydrogels resisted the gravity of 50 g and were repeatedly processed into “circle”, “square”, and “triangle” shapes without any disturbance on fluorescence emission, judging the excellent shaping ability (Figure 3F). The maximum tensile stress was measured to be 7.14 KPa with tensile strain of 160％ (Figure 3H), which showed initial rise followed by a decline, attributable to 3D network structure formed by physiochemical crosslink and subsequent fracture, and could be regulated via changing the usage amount of borax (Figure S17, Supporting Information). High‐adhesion property was also concluded via making the dual‐color hydrogel being adhered onto the surfaces of balance weight, glass, wood, polyethylene (PE) and clothing (Figure 3D), and steadily picking up them with the peel strength of ≈1.837 N (Figure S18, Supporting Information), and at the same time, the fluorescence emission color insignificantly changed. Besides, the hydrogel was tightly pasted onto the surface of finger joint no matter it was straight or bent, revealing the smart interface self‐adaptability (Figure 3E). According to scanning electron microscopy (SEM) analysis, TPE@Mn@ZnS@AG@PVA presented a continuous 3D porous network morphology consisting of curly flakes (Figure 3H), which looked like that of hydrogel alone (Figure S19, Supporting Information) and was conductive for target sufficiently migrating inside hydrogel, suggesting *b‐*TPE NPs and *r‐*Mn@ZnS QDs gave rise to little effect on microstructure. Additionally, high transmittance >95％ in the range of 300–700 nm was recorded, and then made Chinese characters under hydrogel were clearly observed (Figure 3I), indicating hydrogel matrix had essentially little effect on fluorescence emission of *b‐*TPE NPs and *r‐*Mn@ZnS QDs. Further, the water contact angle rapidly declined from 58.4 to 10.9° with lengthy time (Figure 3J), which justified the excellent interface hydrophilicity not only allowing target analyte to sufficiently diffuse but also producing a strongly bound hydration layer for improving the anti‐interference ability.

**Figure 4:**
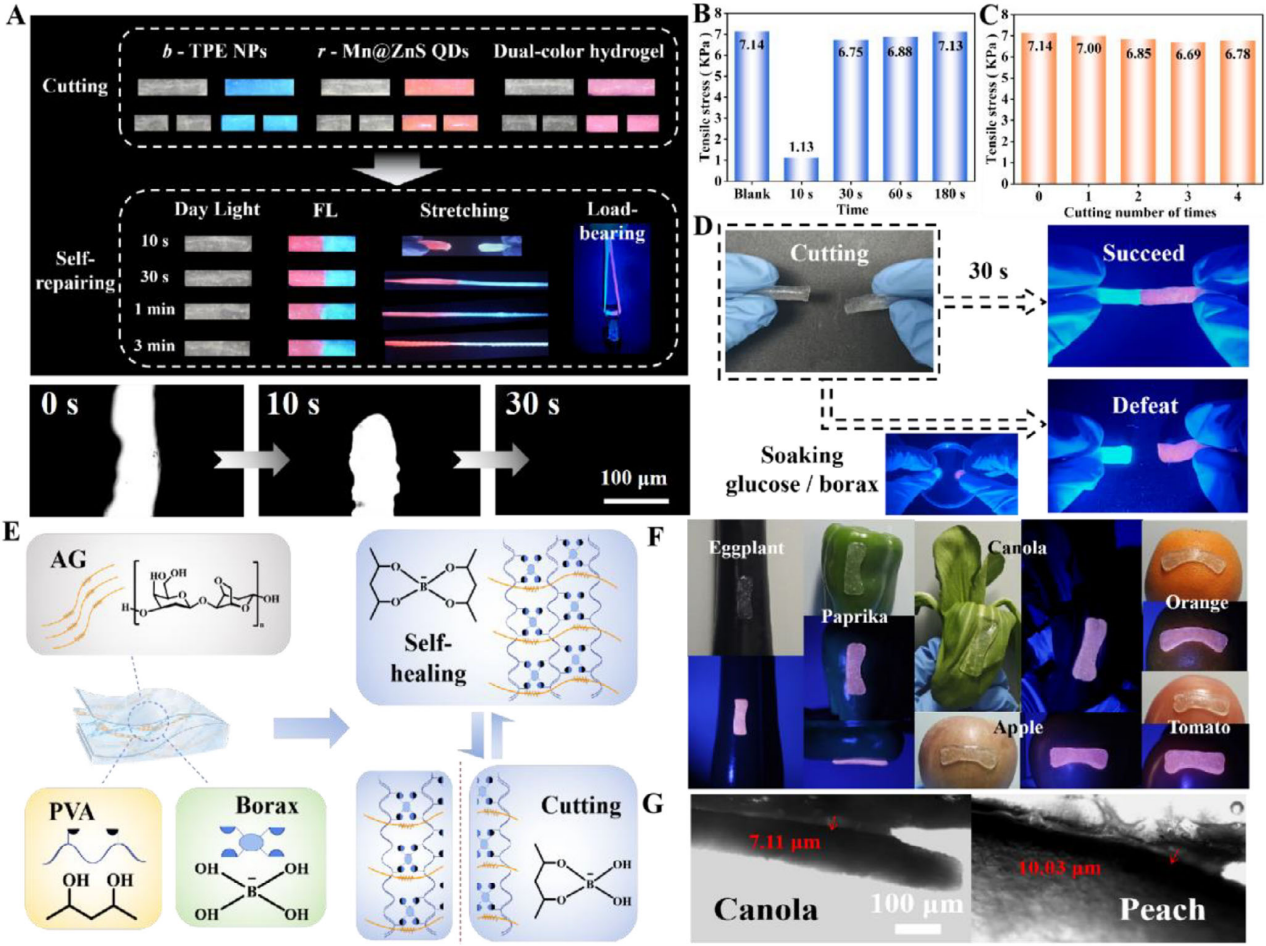
A) Daylight and fluorescence images of cut (top) and self‐repairing hydrogels (middle), and interval distance characterizations of two hydrogels versus contact time (bottom). B) Tensile stress of TPE@Mn@ZnS@AG@PVA exposing to different contact time. C) Tensile stress of TPE@Mn@ZnS@AG@PVA exposing to different cutting number of times. D) The disturbance of glucose/borax on self‐repairing function. E) The sketch map of self‐repairing mechanism of TPE@Mn@ZnS@AG@PVA. F) Daylight and fluorescence images of TPE@Mn@ZnS@AG@PVA being pasted onto different agricultural products. G) Cross‐sectional images of TPE@Mn@ZnS@AG@PVA and skin of peach/leaf of bok choy.

Notably, the as‐prepared TPE@Mn@ZnS@AG@PVA showed preeminent self‐repairing function, which was characterized in **Figure** **4**A–C via first cutting off and subsequently combining at different contact times. The re‐combined fluorescent hydrogel could be easily tore at the time of 10 s with maximum tensile stress of only 1.13 KPa (Figure 4B). Prolonging the time to be 30, 60, and 180 s, respectively, both the hydrogels were firmly united to resist the stretch with tensile stress of 6.75, 6.88, and 7.13 KPa, which were approximately the same as that of uncut hydrogel. It was not surprise to us that longer time was favorable to make both hydrogels interact sufficiently, which was proven by the gradually narrowed until disappeared split (bottom in Figure 4A). Similar variation could also be observed for blue‐emission and red‐emission hydrogels (Figure S20, Supporting Information). Interestingly, *b‐*TPE NPs/*r‐*Mn@ZnS QDs could not diffuse from one hydrogel to the other hydrogel, which implied the high stability of dual‐color hydrogel due to the shielding effect. In addition, more cutting number of times at the same position also did not affect the tensile stress of re‐combined hydrogels (Figure 4C). All these information suggested TPE@Mn@ZnS@AG@PVA featured outstanding self‐repairing function. To gain deep insight into the self‐repairing mechanism, both the cut hydrogels were first soaked in glucose/borax solution and subsequently combined together. Unfortunately, glucose/borax‐treated hydrogels could not be united together (Figure 4D). On the basis of these information, the self‐repairing mechanism was anticipated as below: the cut hydrogels exhibited abundant surface boronic acid and cis‐diol groups, and hence when they were re‐contacted, high‐affinity interaction between boronic acid and cis‐diol was switched, then achieving the self‐repairing (Figure 4E). From this opinion, it was not surprise to us that glucose/borax‐treated hydrogels did not show self‐repairing function because of the same surface group‐inhibited high‐affinity reaction between boronic acid and cis‐diol. According to negligibly variable fluorescence color and RGB values (Figure S21, Supporting Information), the dual‐color hydrogel fascinated high durability, strong anti‐photo‐bleaching property and high resistance to moisture for long‐term preservation. Further, *b*‐TPE NPs and *r*‐Mn@ZnS QDs were tightly encapsulated inside AG‐PVA‐borax‐assembled hydrogel without no any leakage into the solution (Figure S22, Supporting Information), meaning TPE@Mn@ZnS@AG@PVA displayed sufficiently stability and could be used in water environment for long time. Profiting from the big‐league flexibility, TPE@Mn@ZnS@AG@PVA was tightly pasted onto the interfaces of apple, orange, eggplant, paprika, bok choy and tomato with insignificantly changed fluorescence color and strength (Figure 4F), and the slit was respectively measured to be only 7.11/10.03 µm between hydrogel and skin of peach/leaf of bok choy (Figure 4G), indicating the high compactness and superior adhesion toward plant tissues. All the above information suggested TPE@Mn@ZnS@AG@PVA featured towering interface self‐adaptability, and thus could be tightly pasted onto any surfaces of plant tissues, driving the undamaged acquisition of pesticide residue information from living crops.

### 2.4 Analytical Performance of Dual‐Color Hydrogel

**Figure 5:**
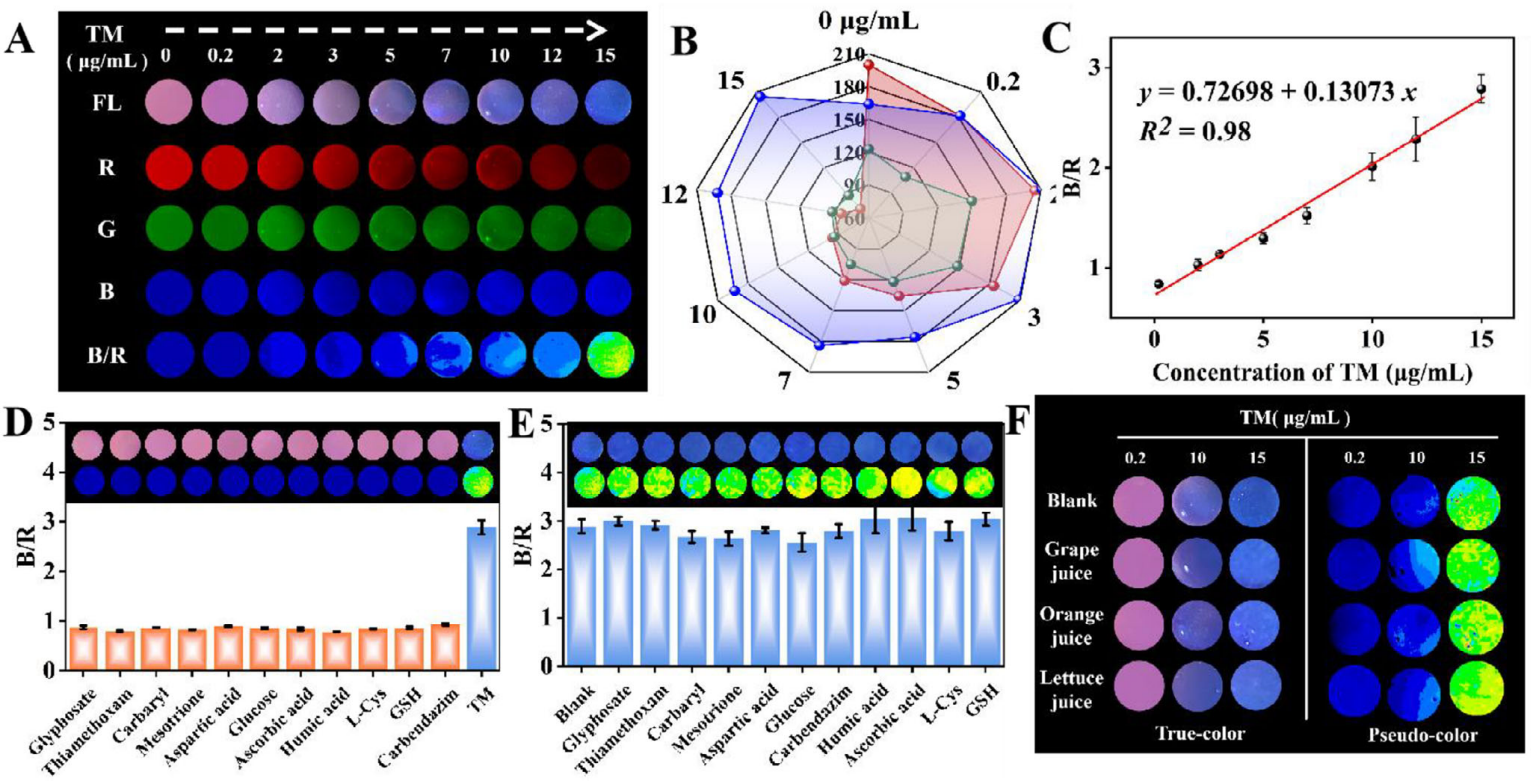
A) Daylight, fluorescent, R, G, B, and B/R pseudo color images of TPE@Mn@ZnS@AG@PVA in the presence of TM with changed amount. B) Relationship between R, G, and B intensity and TM amount. C) Linear relationship between B/R response and TM amount. D) Selective characterization of TPE@Mn@ZnS@AG@PVA. E) Anti‐interference performance of TPE NPs‐Mn@ZnS QDs toward TM in the presence of different interfering substances. Blank indicated the presence of TM alone. F) Pseudo color images of TPE@Mn@ZnS@AG@PVA toward crop extract containing TM with changed amounts.

Dual‐color hydrogel TPE@Mn@ZnS@AG@PVA with circular shape (10 mm diameter and 0.4 mm thickness) was served as fluorescence patch for quantitative detection of TM based on target‐induced fluorescence color evolution and intelligent signal processing. Upon the dropping of TM solution, it exhibited a rapid dynamic diffusion equilibrium to penetrate throughout the double‐network hydrogel, and then maximum collided with *r*‐Mn@ZnS QDs to quench the red‐channel fluorescence, contributing to dual‐color detection of TM. **Figure** **5**A gave the fluorescence photographs of patch subjected to TM with variable concentrations. Expectedly, the introduction of TM weakened red‐channel fluorescence, and with TM concentration increasing, red‐channel fluorescence signal became weaker and weaker but keeping insignificant blue‐channel fluorescence variation. Aiming to realize quantitative analysis, the fluorescence colors were split into R, G, and B images. Obviously, the color intensity of R image weakened with increasing TM concentration, and in contrast, B image intensity positively varied, keeping pace with RGB values variation in Figure 5B. Further, we made B/R to get pseudo‐color images for higher‐sensitive detection of TM. The data in Figure 5C suggested the as‐prepared dual‐color patch depicted a working equation of B/R = 0.726+0.131C_TM_ in the linear range of 0.2‐15 µg mL^−1^ with a co‐efficient of 0.98, and the LOD was calculated to be 0.045 µg mL^−1^ based on 3σ rule, which was lower than the legal limit stipulated by Chinese National Food Safety Standard (GBT‐2763‐2021). Taken together, the as‐fabricated hydrogel patch used color information as output signal instead of peak intensity to achieve cost‐effective, rapid and visual detection of TM without professional technicians and large‐scale/expensive apparatuses, delivering a powerful alarm tool for on‐site monitoring pesticide residue.

Further, glyphosate, thiamethoxam, carbaryl, mesotrione, aspartic acid, glucose, GSH, humic acid, L‐Cys, ascorbic acid and carbendazim were used as negative controls to evaluate the detection selectivity. As illustrated in Figure 5D, reddish purple and deep blue for fluorescence and pseudo color aligning with that of blank sample were respectively observed for interfering substances, and only TM impelled the emergence of blue fluorescence and yellow‐green pseudo color of hydrogel patch accompanied with improved B/R value. Furthermore, mixing one of interfering substances with TM did not conspicuously bother the response of fluorescence color, pseudo color and B/R value toward TM alone (Figure 5E). All these information presented TPE@Mn@ZnS@AG@PVA patch enjoyed outstanding characteristic for differencing TM against other interfering substances in complicated system. To gain deep insight into practical usability, the hydrogel patch was explored to detect TM in grape juice, orange juice and lettuce juice via a standard spiking experiment with data in Figure 5F and Table S2 (Supporting Information). The fluorescence and pseudo color images of patches in juice samples exposing to TM with the concentrations of 0.5, 10, and 15 µg mL^−1^ were highly consistent with those of buffer. According to B/R‐based quantitative analysis, the mean recoveries were calculated to be 102.5%/95.4%/96.5%, 94%/96.7%/95.9%, and 110%/95.7%/94.9%, respectively, with all RSDs smaller than 5.7%, suggesting the high repeatability and good accuracy. Further, seven differently prepared hydrogel patches fought 15 µg mL^−1^ TM analysis on almost identical readout signals, implying the high precision (Figure S23, Supporting Information). Such high performance might be ascribable to hydrogel's double‐network structure and anti‐interference ability allowing for pre‐concentration of analyte, and unique recognition efficiency of *r‐*Mn@ZnS QDs to target permitting for a significant quenching in red fluorescence in the presence of only a small number of TM.

### 2.5 Minimally‐Destructive Pesticide Residue Management

**Figure 6:**
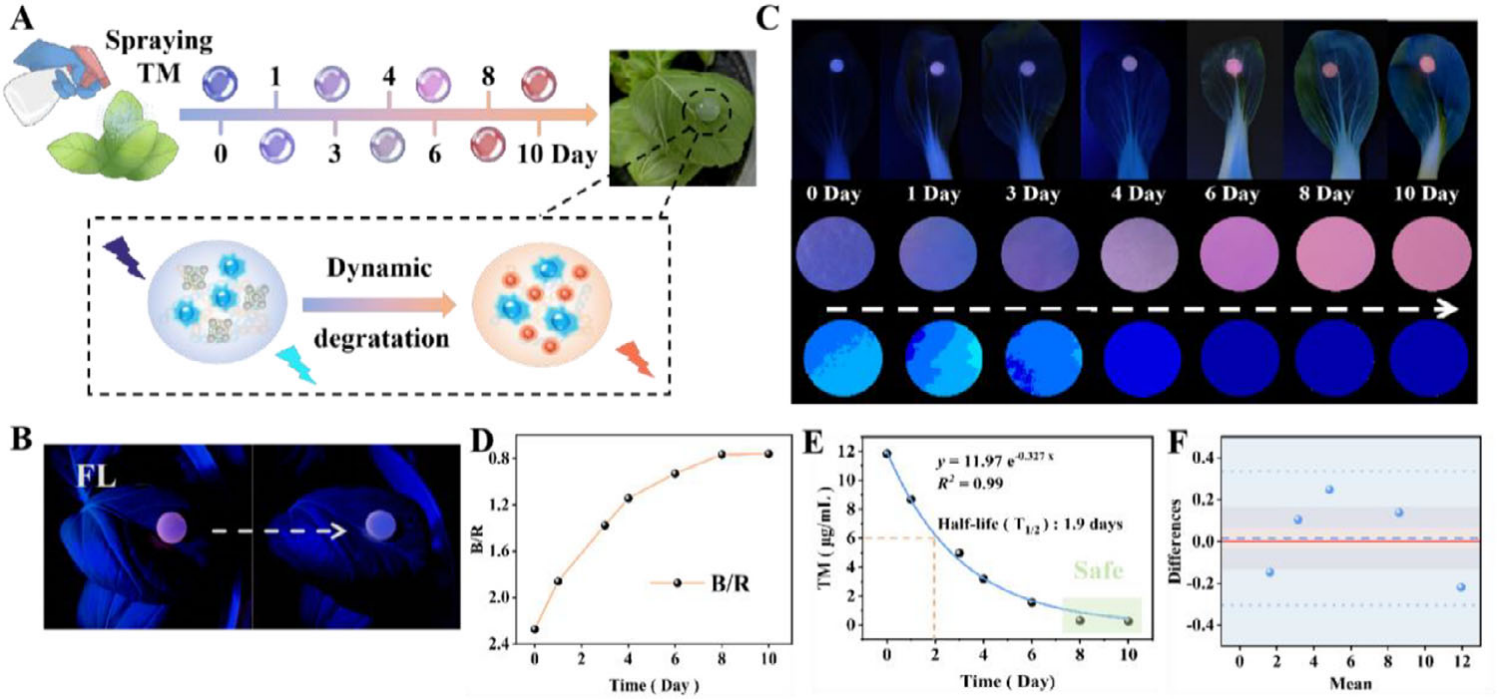
A) Sketch map of profiling the dynamic TM degradation in bok choy by TPE@Mn@ZnS@AG@PVA. B) Fluorescence images of TPE@Mn@ZnS@AG@PVA being pasted onto the surface of bok choy sprayed by TM (right) and no TM (left). C) Fluorescence and pseudo‐color images C), B/R D), and TM concentration E) of TPE@MN@ZnS@AG@PVA being pasted onto the surface of bok choy versus different interval days. F) Bland‐Altman plot of test result correlation between HPLC and our sensor.

There are a lot of sensors reported in sensitively and accurately reporting pesticide residue information, but crop plants are required to be crushed for extracting pesticide residue, and thus resulted in complicated operation, lengthy analytical time and technical personnel, making detrimental to POC application. Aiming to overcome such issues, the dual‐color hydrogel TPE@Mn@ZnS@AG@PVA patch was attempted to be pasted onto the surface of leaf of bok choy for minimally‐destructive management of TM residue. Moreover, the leaf surface microenvironment did not affect the fluorescence emission of *b*‐TPE NPs and *r*‐Mn@ZnS QDs (Figure S24, Supporting Information). The bok choy was first sprayed by TM solution (commercial product) with content of 1.5%, and the upper surface of leaf was treated by micro‐needles (Figure S25, Supporting Information) at different interval time for promoting the systemic TM to migrate, and immediately after hydrogel patch was pasted onto the surface of micro‐needle‐punctured leaf, where interfacial and interior TM diffused into the hydrogel to yield different fluorescence color response, consequently achieving quantitative detection in minimally‐destructive manner (**Figure** **6**A). As anticipated, the hydrogel patch exhibited bright reddish purple‐emission fluorescence for bok choy without spray by TM, and in stark contrast, obvious blue‐emission fluorescence of patch was surveyed for TM‐sprayed bok choy (Figure 6B), and the similar fluorescence color response was also recorded for tomato /grape without or with spray by TM (Figure S26, Supporting Information), in accordance with detection principle that TM residue migrated into hydrogel to silence the red‐channel fluorescence of *r‐*Mn@ZnS QDs while maintaining negligible variation in blue‐channel fluorescence of *b‐*TPE NPs. Meanwhile, after storing for 72 h, TPE@Mn@ZnS@AG@PVA also displayed similar fluorescence response to bok choy treated by TM or no TM (Figure S27, Supporting Information), indicating the outstanding long‐period durability. Further, with extended interval days, the red‐channel fluorescence strength of patch was gradually improved to induce the evolution from blue to reddish purple, along with the gradual pseudo color transformation from sky‐blue to deep blue (Figure 6C), which indicated the gradually reduced TM amount in bok choy, suggesting the dynamic degradation. The B/R value also declined versus the lengthy interval day within expectation (Figure 6D). According to working equation, the relationship between concentration of TM (y) and interval time (x) was investigated in Figure 6E. TM degradation in bok choy followed the pseudo‐first‐order kinetics with equation of y = 11.97e^−0.327x^, and tracking during the 10‐day interval time revealed the half‐life of 1.9 days and safety period since the 8^th^ day after spraying TM. Similar test results could also be received from TPE@Mn@ZnS@AG@PVA being pasted onto the surface of leaves of other batch of bok choy (Figure S28, Supporting Information). Moreover, the test results displayed a high correlation with that of HPLC assay according to Bland‐Altman analysis of 95% limit of agreement and paired *t*‐test data with *p* = 0.997 and σ = 0.168 (Figure 6F), undoubtedly justifying the excellent practical usability of TPE@Mn@ZnS@AG@PVA platform. Taken together, the TPE@Mn@ZnS@AG@PVA patch mounted onto the surface of leaf of bok choy successfully in situ profiled the dynamic degradation process of TM in a minimally‐destructive manner, and thus rendered an important tool to judge the harvesting period of agricultural crops, guaranteeing the food safety and human health.

## 3 Conclusion

In summary, we have constructed *b‐*TPE NPs and *r‐*Mn@ZnS QDs‐co‐embedded dual‐color hydrogel as a flexible wearable sensor for in situ and minimally‐destructive reporting pesticide residue information from living crops. Well‐defined *b‐*TPE NPs with no‐response to TM performing blue fluorescence, and *r‐*Mn@ZnS QDs exhibiting red fluorescence and selective yet direct response to TM, served as fluorescence indicators to be encapsulated into PVA, borax and AG for constructing the double‐network hydrogel TPE@Mn@ZnS@AG@PVA fascinating excellent self‐repairing function, smart interface self‐adaptability, high adhesion and big‐league flexibility. TM diffused inside the hydrogel to quench the red fluorescence while maintaining blue fluorescence negligible variation, which then drove fluorescence color to gradually evolve from reddish purple to blue, contributing to quantitative detection of TM with LOD down to 0.045 µg mL^−1^ via splitting fluorescence image into RGB color mode and intelligent pseudo color signal processing. Such unique properties further enabled TPE@Mn@ZnS@AG@PVA patch to be pasted onto the surface of vegetables and fruits, and thereby to achieve TM residue minimally‐destructive monitoring, profiling the dynamic degradation in bok choy through recording residue information at variable time points. This work pioneered the advanced developments of dual‐color hydrogel as wearable crop sensor and rendered a novel paradigm for in situ and on‐site monitoring pesticides residues in living crops with minimal destruction.

## 4 Experimental Section

### Preparation of *r*‐Mn@ZnS QDs

0.43 g ZnSO_4_·7H_2_O, 0.363 g L‐Cys and MnCl_2_·4H_2_O with varying amounts were dissolved in 75 mL of water. 1.0 m NaOH solution was applied to adjust pH value to 11, and immediately after 15 mL of Na_2_S solution (0.2 m) was added drop by drop. The reaction solution was heated to 70 °C for 14 h under N_2_ atmosphere. At last, the solution was treated by centrifugation (10 000 rpm, 10 min) to get the supernatant, which was on dialysis for 24 h using a 2000 DA dialysis bag to acquire the resultant *r*‐Mn@ZnS QDs.

### Preparation of *b*‐TPE NPs

0.05 g TPE‐OH was dissolved in 5 mL of THF to get A, and 0.05 g PSMA was dissolved in 5 mL of THF to get B. 800 µL of A was mixed with 200 µL of B, and subsequently the mixed solution was added into 9 mL of water under ultrasound treatment for 15 min. Finally, *b*‐TPE NPs were obtained via centrifugation (12 000 rpm, 5 min) and washed by water (three times), and re‐dispersed in 10 mL of phosphate buffer (PB, 10 mm, pH 7.4) for subsequent application.

### Dual‐Color Detection of TM in Solution

50 µL of *r*‐Mn@ZnS QDs solution, 30 µL of *b*‐TPE NPs solution, 30 µL of solution containing TM with different concentrations and 190 µL phosphate buffer (PB) (10 mm, pH 7.4) were mixed and reacted for specific time. After that, fluorescence spectra were collected under 320 nm light irradiation to evaluate the response of TPE NPs‐Mn@ZnS to TM on the basis of the variation in signal intensity.

### Construction of TPE@Mn@ZnS@AG@PVA

0.05 g AG and 0.4 g PVA were dissolved in 4.0 mL of water by heating, and immediately after 500 µL of *r*‐Mn@ZnS QDs solution and 250 µL of *b*‐TPE NPs solution were added, and the mixed solution was sufficiently shaken until homogeneous dispersion. After that, 1.0 mL of borax aqueous solution (0.04 mm) was quickly injected, and the mixed solution was stirred for 30 min. Finally, the reaction solution was poured into the mould to get dual‐color hydrogel TPE@Mn@ZnS@AG@PVA through cooling.

### Detection of TM Using TPE@Mn@ZnS@AG@PVA

300 µL of TM solution with different concentration was dropped onto the surface of TPE@Mn@ZnS@AG@PVA in two portions. After reacting, the fluorescence images were collected by smart phone in dark environment, and the fluorescence colors were segmented into R, G, and B images. On account of the variation in R/B, the analytical performance of TPE@Mn@ZnS@AG@PVA toward TM was evaluated.

### Minimally‐Destructive Detection of TM in Bok Choy

Bok choy were sprayed by 20 mL of TM solution with the content of 1.5%. After that, the leaf of bok choy was treated by micro‐needles at different interval time (1, 3, 4, 6, 8, and 10 days). Immediately, hydrogel patch TPE@Mn@ZnS@PVA@AG was pasted onto the surface of micro‐needle‐punctured leaf, and 300 µL of mixed solvent comprising of CH_3_OH and buffer was dropped onto the surface of patch in two portions. After reacting for 40 min, the fluorescence photographs were collected and split into R, G, and B values, where the ratio between R and B was used to quantitatively determine the TM residue.

## Conflict of Interest

The authors declare no conflict of interest.

## Supporting information



Supporting Information

## Data Availability

The data that support the findings of this study are available from the corresponding author upon reasonable request.
