# Supplementary material for: Interface‐Adaptive Dual‐Color Hydrogel with Self‐Repairing Function and High Adhesion as Flexible Wearable Sensor for Minimally‐Invasive Monitoring Pesticide Residue in Living Crop
Source: Adv Sci (Weinh). 2025 Oct 13;13(2):e12386. doi: 10.1002/advs.202512386 (PMC12786363; doi:10.1002/advs.202512386)
Supplement: Supplementary file 1 — Supporting Information [file ADVS-13-e12386-s001.docx]

Copyright WILEY-VCH Verlag GmbH & Co. KGaA, 69469 Weinheim, Germany, 2016.

Supporting Information

**Interface-Adaptive Dual-Color Hydrogel with Self-Repairing Function and High Adhesion as Flexible Wearable Sensor for Minimally-Invasive Monitoring Pesticide Residue in Living Crop**

Jianling Chen^ab^, Zihan Zhu^a^, Yafei Li^a^, Zizhe Wang^a^, Wendong Wang^b^, Hong Chen^c^, Xuelian Xin^a^, Zhenguo Chi^d^, Haiyin Li^ab*^

# Experimental Section

**Apparatus.** Ultraviolet-visible spectra were characterized on a UV-1900 spectrometer (Shimadzu, Japan). RF 6000 fluorescence spectrophotometer (Shimadzu, Japan) was explored to characterize the luminescence property of prepared *r*-Mn@ZnS QDs and *b*-TPE NPs. SEM (JEOL, Japan) and TEM (FEI, USA) were employed to observe the morphological structure. Fourier transform infrared spectroscopy (FT-IR) was carried out on a Thermo Field Nicolet iS10 infrared spectrometer (USA). Zeta potential was measured using a ZS90 nanometer particle size potentiometer (Malvern, UK). The contact angle was collected by contact angle instrument OCA 15EC (Dataphysics, Germany). The slit was characterized by microscope (Nikon, Japan). The mechanical properties of hydrogels were tested by tensile testing machine (DANA, China). HPLC data were recorded using Thermo Feld QE Focus HPLC (Thermo, USA) to verify the test accuracy. XPS characterizations were conducted on Thermo Scientific EscaLab 250Xi (Thermo, USA) for examining the element compositions. Raman spectrum was characterized by LabRAM HR Evolution (HORIBA, France). The test conditions for acquiring the fluorescence images of TPE@Mn@ZnS@AG@PVA were listed as below: all operation were carried out in a dark box (HYAN400-400, Jingna technology, China) with size of 400×400×350 cm; the light source with power of 12 W and wavelength of 310 nm (UVGO, China) was used to excite TPE@Mn@ZnS@AG@PVA for obtaining the fluorescence images; the light source was positioned above the hydrogel with distance of about 10 cm on the left while the HUAWEI P60 phone was positioned above the hydrogel with distance of 10 cm on the right to get the fluorescence images.

**Materials.** All of the reagents with analytical grade were purchased and used without further processing required. Glyphosate, thiamethoxam, mesotrione, carbaryl, glucose, L-cysteine (L-Cys), MnCl_2_·4H_2_O, Na_2_S·9H_2_O and ZnSO_4_·7H_2_O were bought from Shanghai Energy Chemical Co., Ltd (Shanghai, China). Thiophanate-methyl (TM) was purchased from Shanghai Yuanye Bio-Technology Co., Ltd (Shanghai, China). Polyvinyl alcohol 1788 (PVA) was purchased from Tianjin Huasheng Chemical Reagent Factory (Tianjin, China). Agarose was purchased from BBI Life Sciences Co., Ltd (Hong Kong, China). Tetrahydrofuran (THF) was purchased from Tianjin Kemiou Chemical Reagent Co., Ltd.(Tianjin, China). NaOH, TPE-OH and PSMA were purchased from Shanghai Titan Scientific Co., Ltd (Shanghai, China). Methanol was purchased from Tianjin Kermel Chemical Reagent Co., Ltd (Tianjin, China). Aspartic acid, glutamic acid, borax and glutamate were purchased from Aladdin Reagent Co., Ltd (Shanghai, China).KCl, NaCl were purchased from Tianjin Fuchen Chemical Reagent Co., Ltd. (Tianjin, China). All the pure water used in this work was originated from Wahaha Business Co. Ltd (Hangzhou, China). The micro-needle patch used in this work has a diameter of 2 cm with the number of 1000, length of 300 µm and tip diameter of 20 µm.

**Table S1** Comparison of our sensor with previously reported sensors for TM in LOD, linear range, and detection time.

| Method | Signal probe | Recognition ion | Linear range | LOD | Time | Ref |
| --- | --- | --- | --- | --- | --- | --- |
| Luminescence | TPE NPs-Mn@ZnS QDs | / | 0.002 ~ 2 μg/mL | 0.054 ng/mL | 2 min | This work |
| Luminescence | CDs@Cu-MOFs | Cu^2+^ | 0.0307~ 0.769 μM | 12 ng/mL | 10 min | 1 |
| Luminescence | CDs/Cu | Cu^2+^ | 0.10 ~ 20.00 μM | 0.991  ng/mL | 5 min | 2 |
| Colorimetry | Fe_3_O_4_/GONRs | Cu^2+^ | 0.02 ~ 10 μg/mL | 28 ng/mL | 10 min | 3 |
| Luminescence | HM-CDs@ZIF-8 | / | 0.00171 ~ 3.4239 M | 692 ng/mL | 12.5 min | 4 |
| Luminescence | Y-G-CDs | / | 0 ~ 10 μM | 17 ng/mL | 30 min | 5 |
| SERS | Fe_3_O_4_@SiO_2_@Ag- SH NP | / | 0.05 ~ 0.6 mg/L | 67 ng/mL | 15 min | 6 |

**Table S2.** Standard addition experiment of the prepared hydrogel in grape, orange and lettuce extract solution.

| **No.** | **Added**  **(μM)** | **Mean measured**  **(μM)** | **Mean recovery ^a^**  **(%)** | **RSD**  **(%)** |
| --- | --- | --- | --- | --- |
| Grape | 0.2  10  15 | 0.205  9.54  14.48 | 102.5  95.4  96.5 | 3.6  5.3  4.1 |
| Orange | 0.2  10  15 | 0.188  9.67  14.39 | 94  96.7  95.9 | 5.7  4.2  4.9 |
| Lettuce | 0.2  10  15 | 0.22  9.57  14.24 | 110  95.7  94.9 | 4.3  4.8  5.5 |


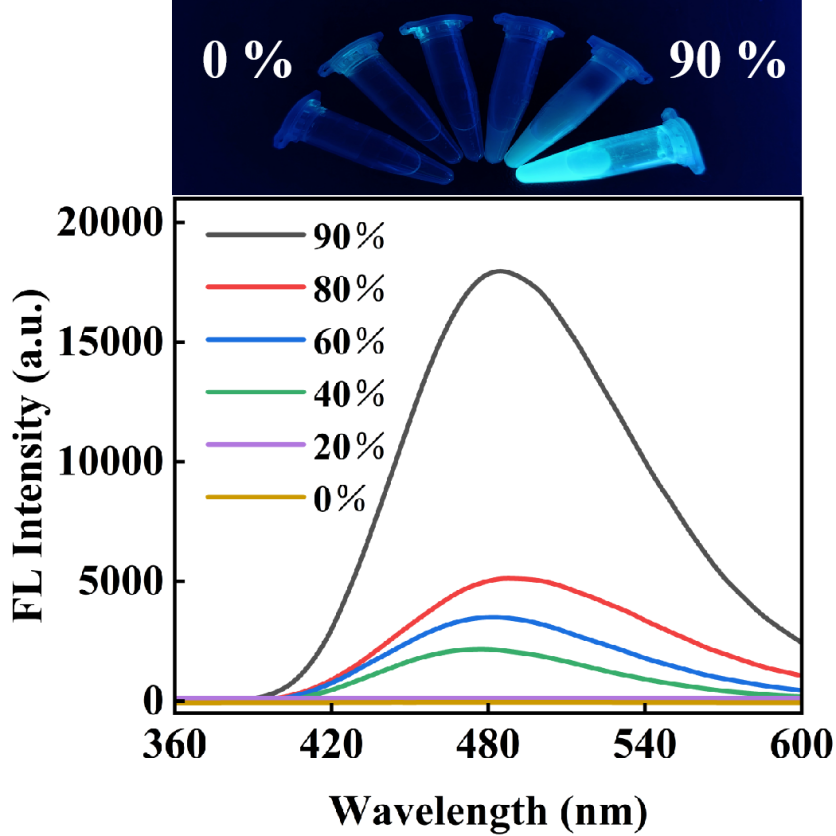


**Figure S1** Fluorescence spectra and images of TPE-OH in THF-H_2_O mixed solvent with different water fraction.


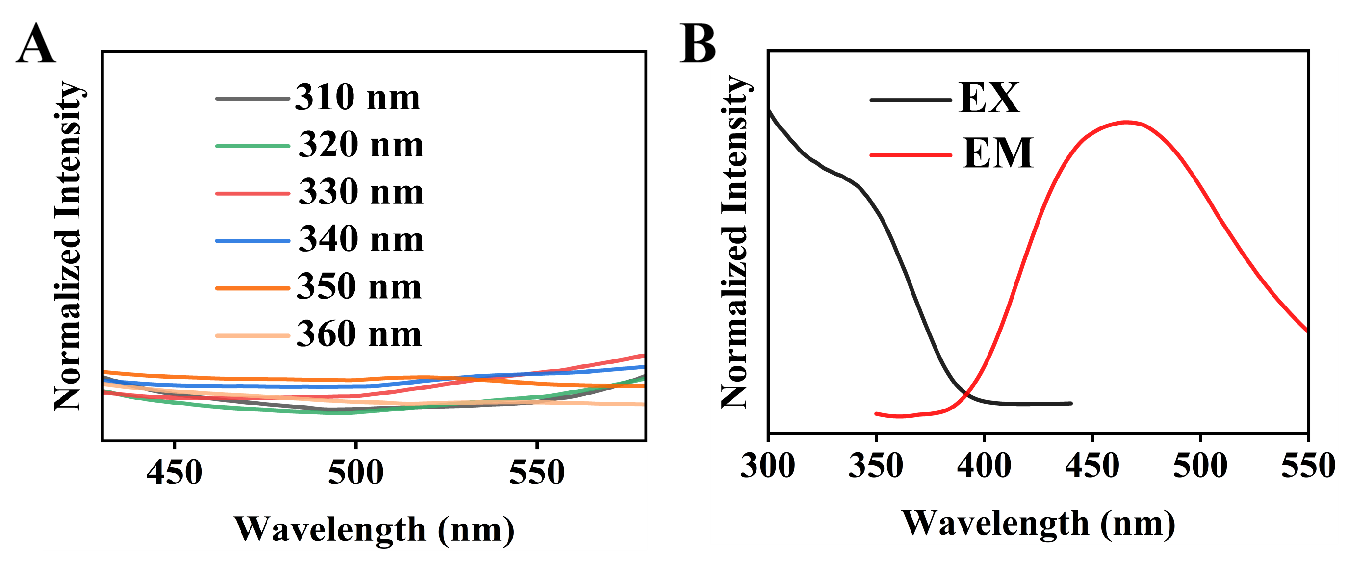


**Figure S2** (A) Fluorescence spectra of PSMA under the excitation of light with different wavelength. (B) Excitation and emission spectra of TPE-OH.


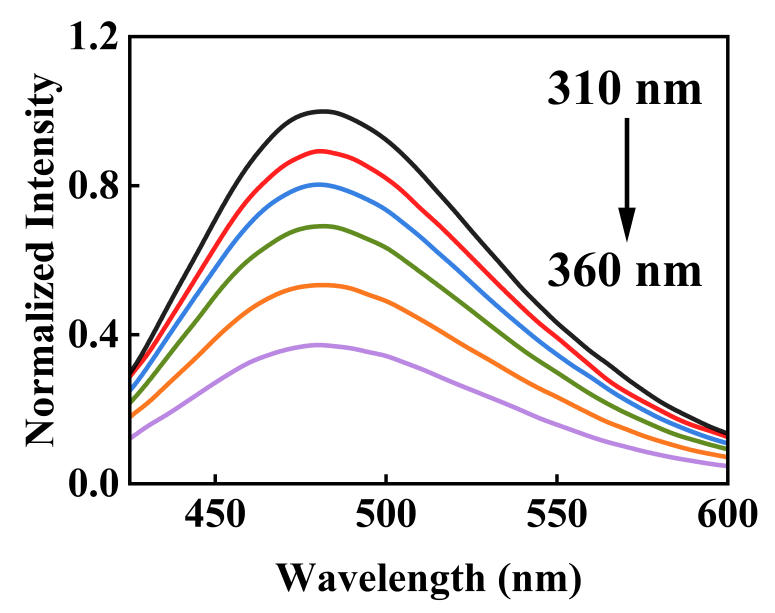


**Figure S3** Fluorescence spectra of *b*-TPE NPs upon the excitation of light with different wavelength.

**
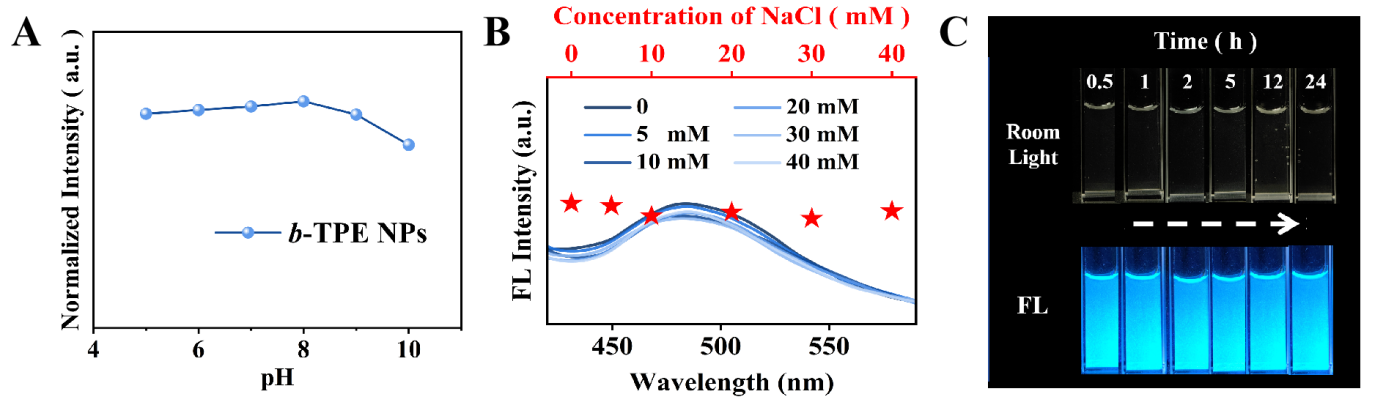
Figure S4** (A) Normalized fluorescence intensity of *b*-TPE NPs in the presence of different pH value. (B) Fluorescence spectra and intensity distribution of *b*-TPE NPs versus different NaCl concentration. (C) Daylight and fluorescence images of *b*-TPE NPs solution after storing for 0.5, 1, 2, 5, 12 and 24 h.


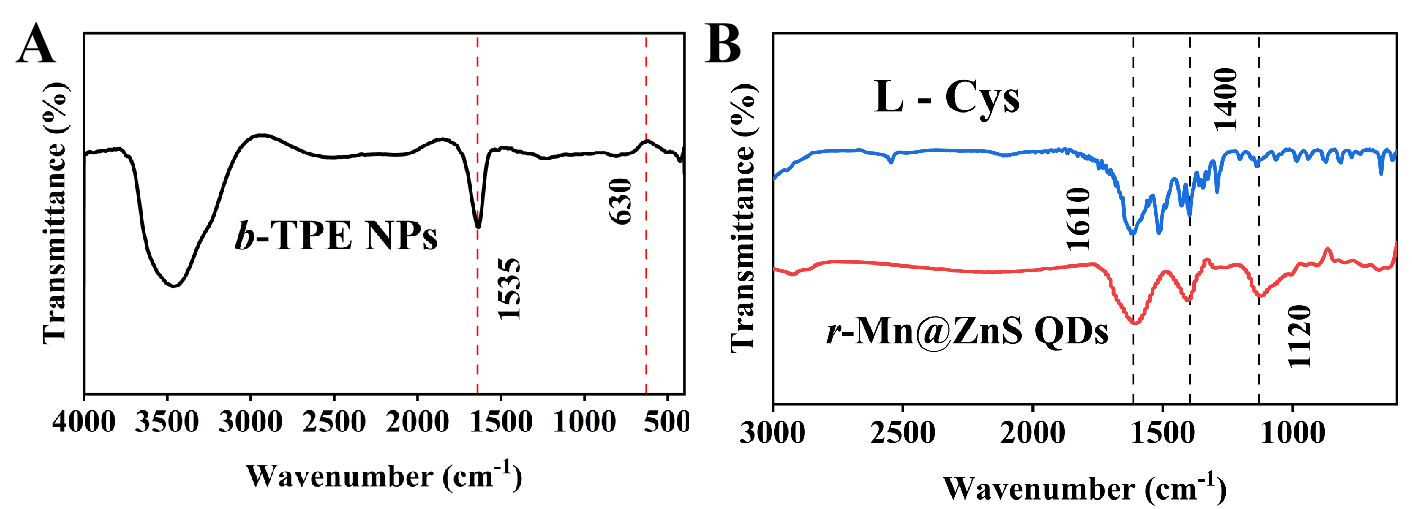


**Figure S5** (A) FT-IR spectra of *b*-TPE NPs. (B) FT-IR spectra of *r*-Mn@ZnS QDs and L-Cys .

**
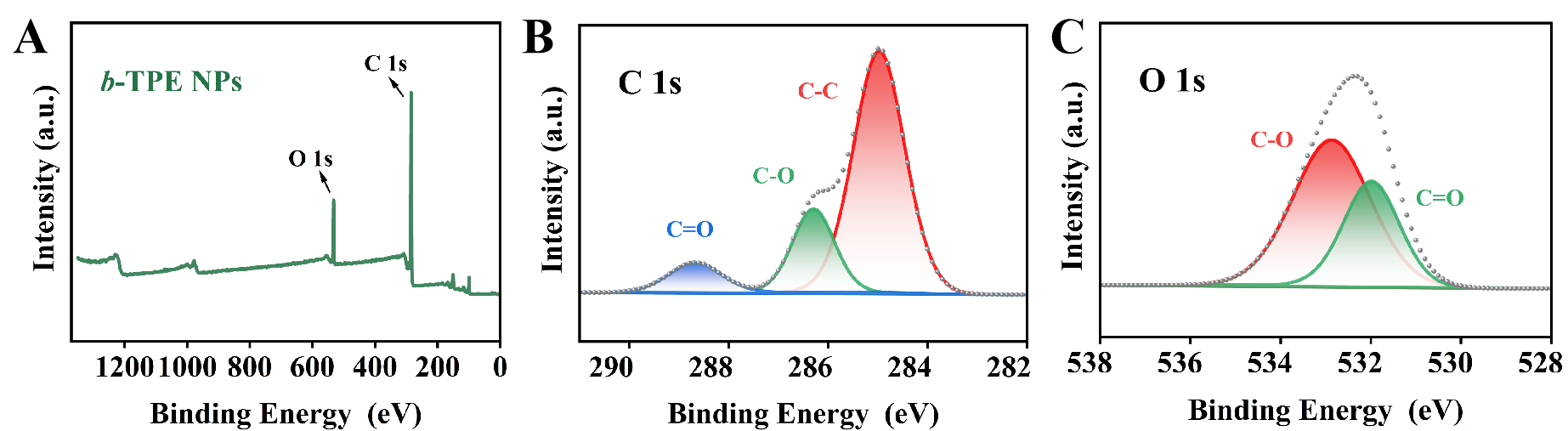
Figure S6** (A) XPS spectrum of *b*-TPE NPs. High-resolution XPS spectra of C 1s (B) and O 1s (C).

**
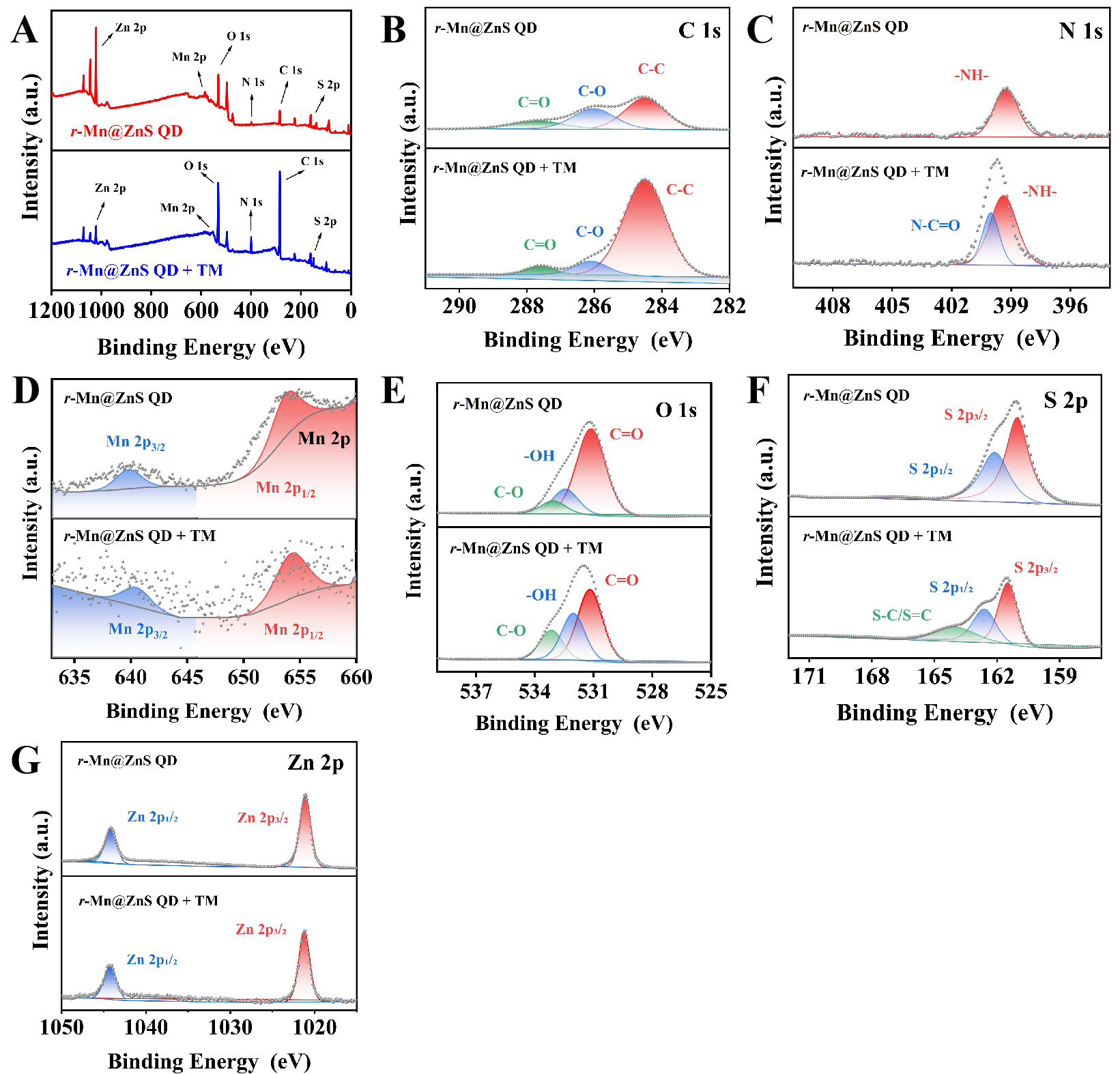
Figure S7** (A) XPS spectrum of *r*-Mn@ZnS QDs. High-resolution XPS spectra of C 1s (B), N 1s (C), Mn 2p (D), O 1s (E), S 2p (F), and Zn 2p (G).


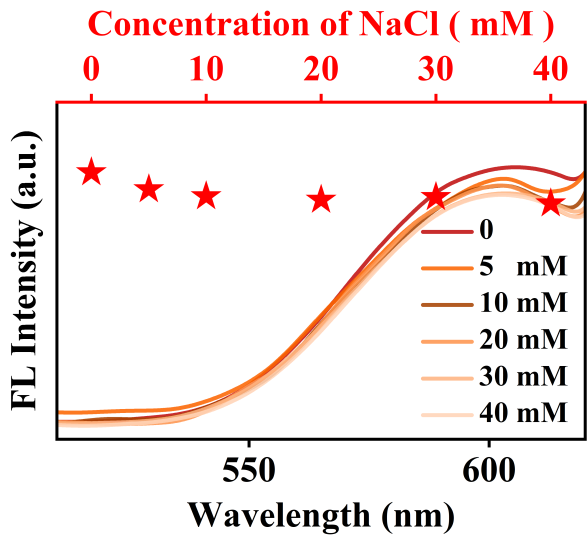


**Figure S8** Fluorescence spectra and intensity distribution of *r*-Mn@ZnS QDs in the presence of different NaCl concentration.


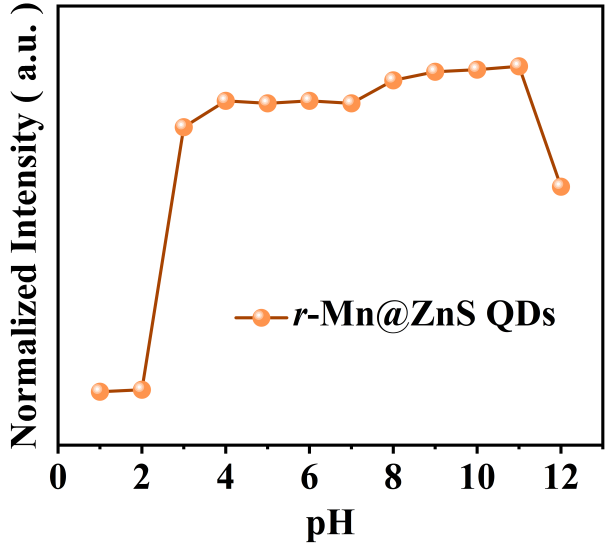


**Figure S9** Fluorescence intensity of *r*-Mn@ZnS QDs in the presence of different pH value.

**
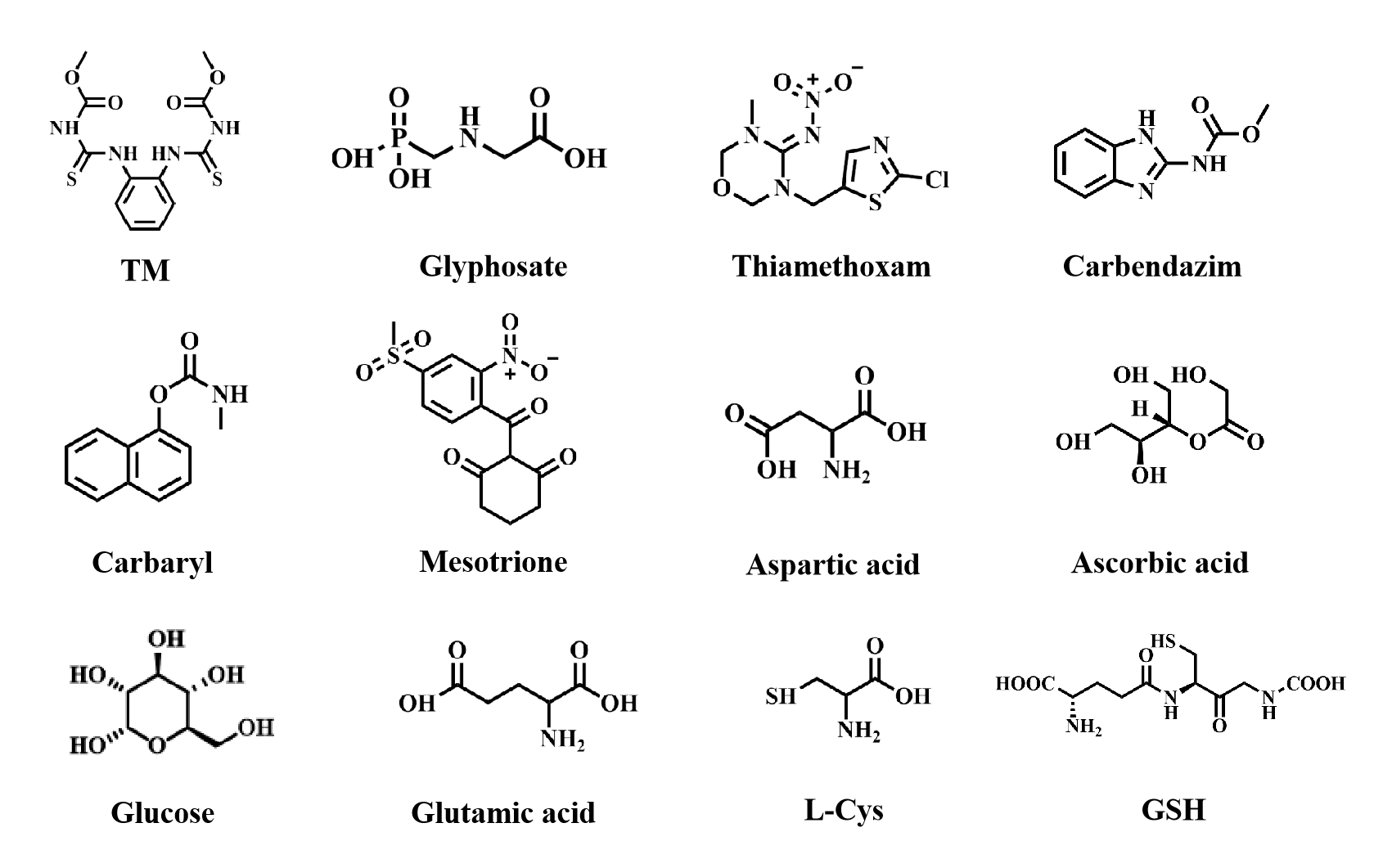
Figure S10** Molecular structures of different analytes.
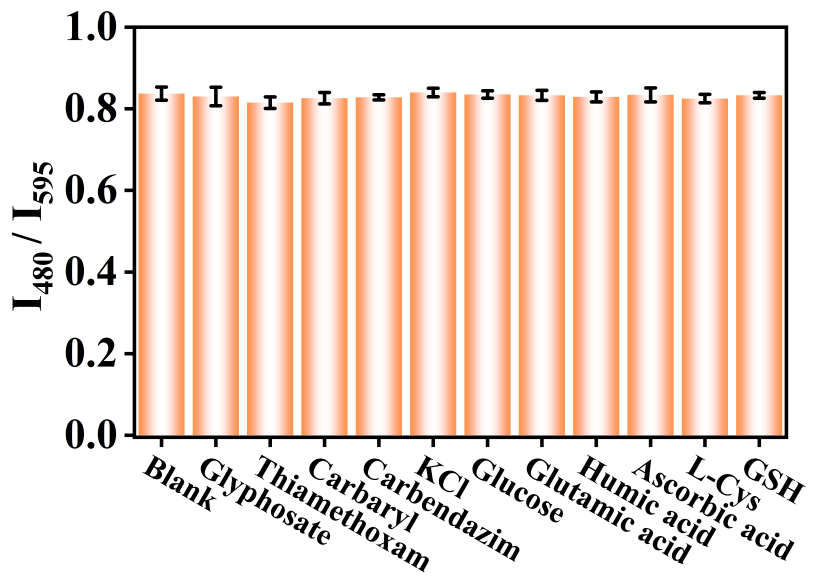


**Figure S11** Anti-interference performance of TPE NPs-Mn@ZnS QDs toward TM in the presence of different interfering substances. Blank implied the presence of only TM.


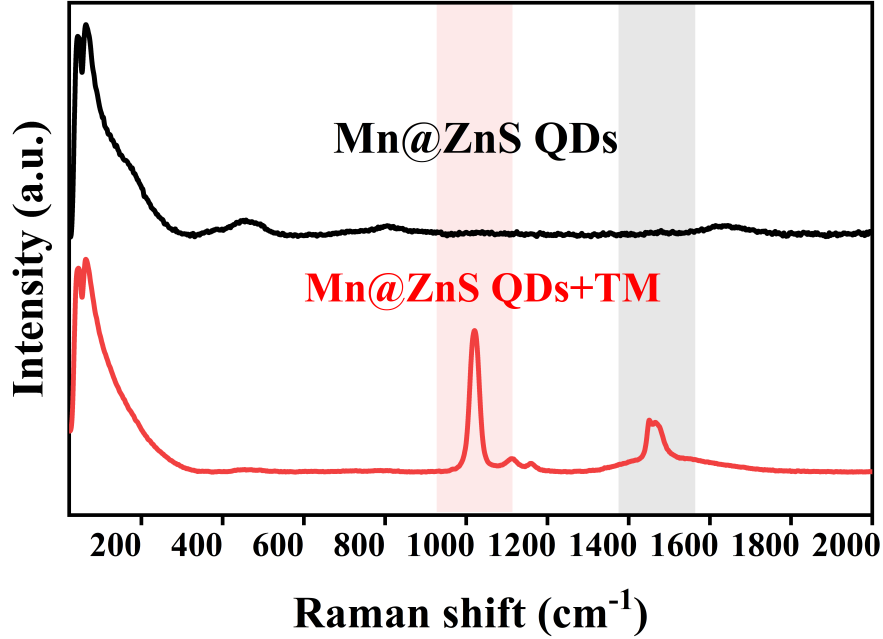


**Figure S12** SERS spectra of *r*-Mn@ZnS QDs and *r*-Mn@ZnS QDs+TM.


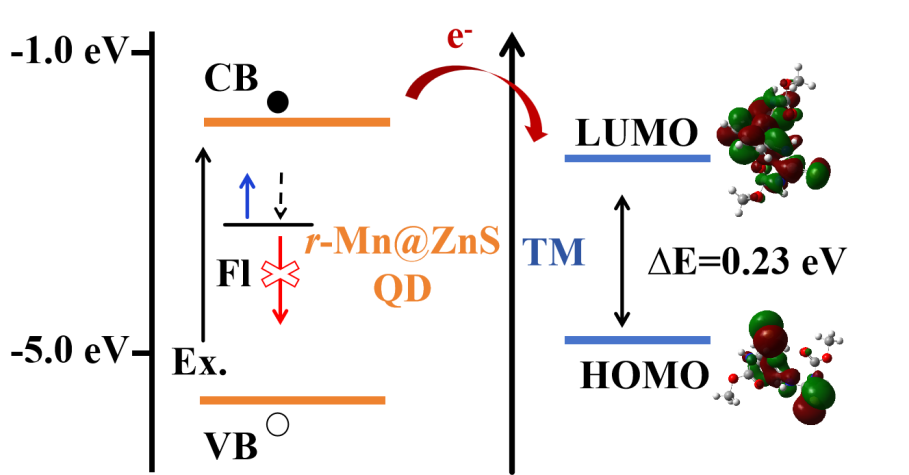


**Figure S13** Theoretical validation of PET from r-Mn@ZnS QDs to TM according to energy level analysis from reported works.^[7, 8]^

**
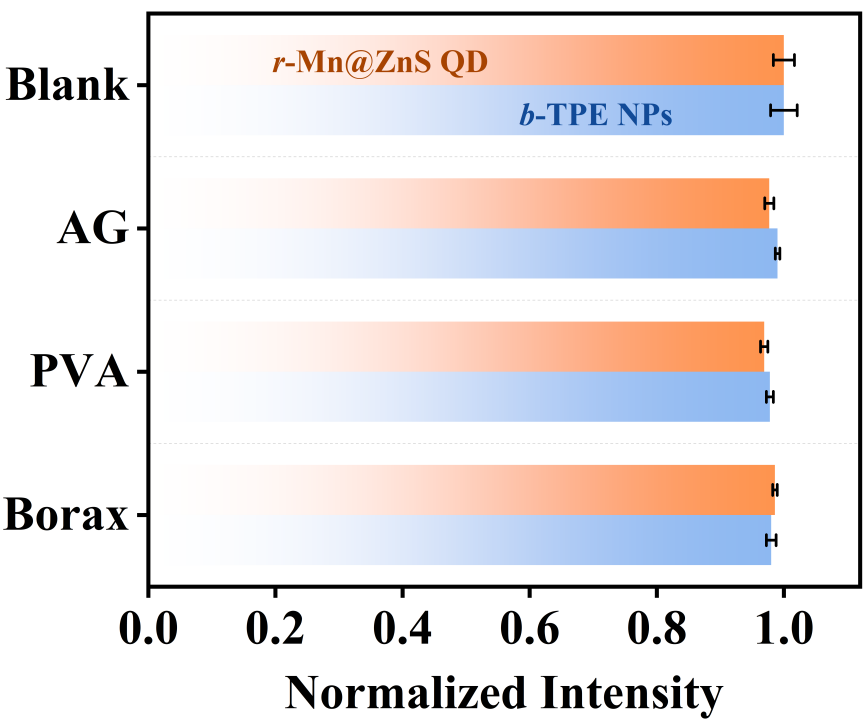
**

**Figure S14** The disturbance of AG, PVA and borax on fluorescence emission of *b*-TPE NPs and *r*-Mn@ZnS QDs.


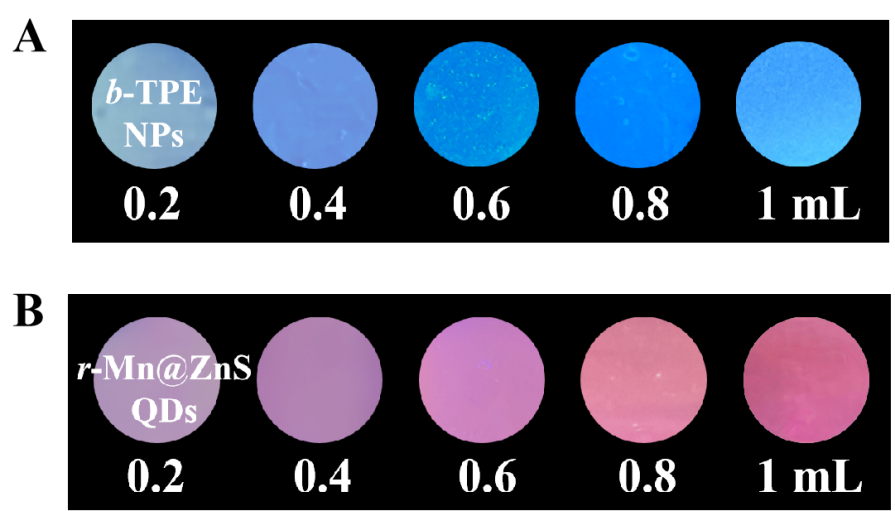


**Figure S15** Fluorescence color of *b*-TPE NPs-involved hydrogel (A) and *r*-Mn@ZnS QDs-involved hydrogel (B) with different volume.


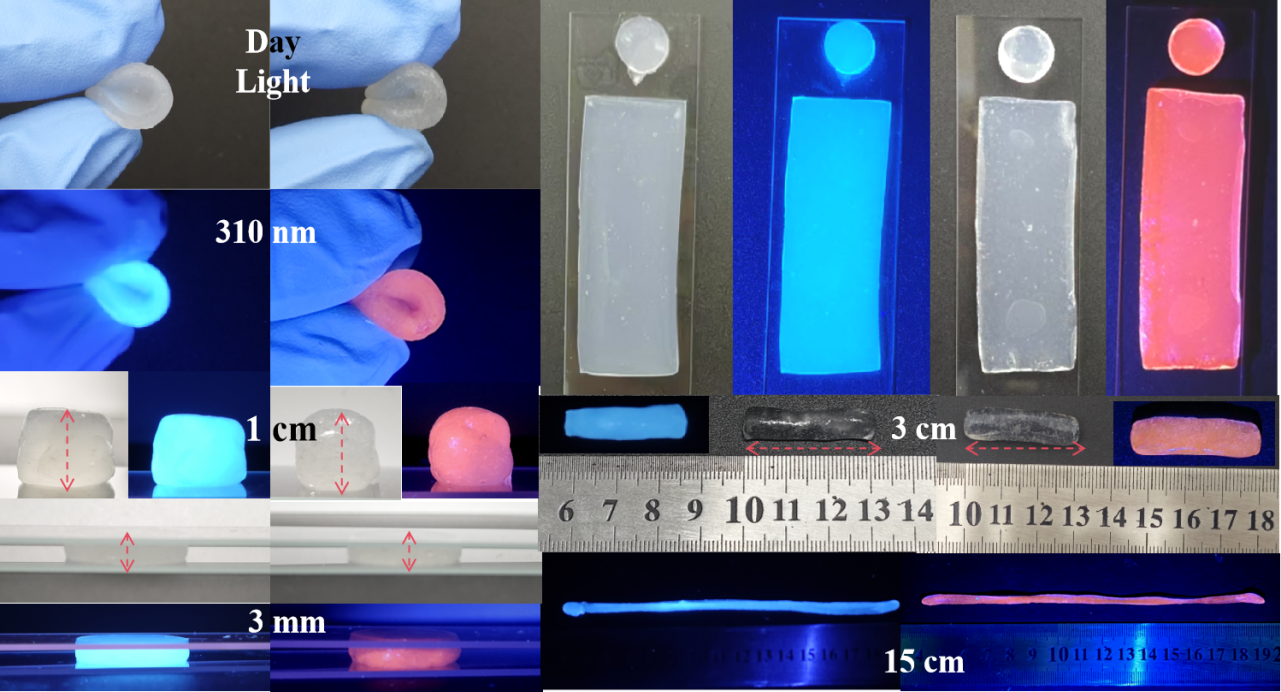


**Figure S16** Daylight and fluorescent hydrogels under the action of compression and stretch.

**
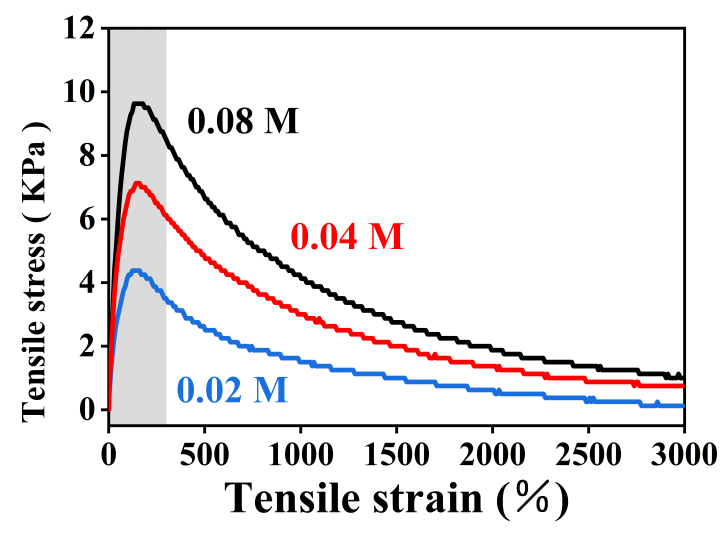
**

**Figure S17** Stress-strain curves of TPE@Mn@ZnS@AG@PVA using different borax amount.

**
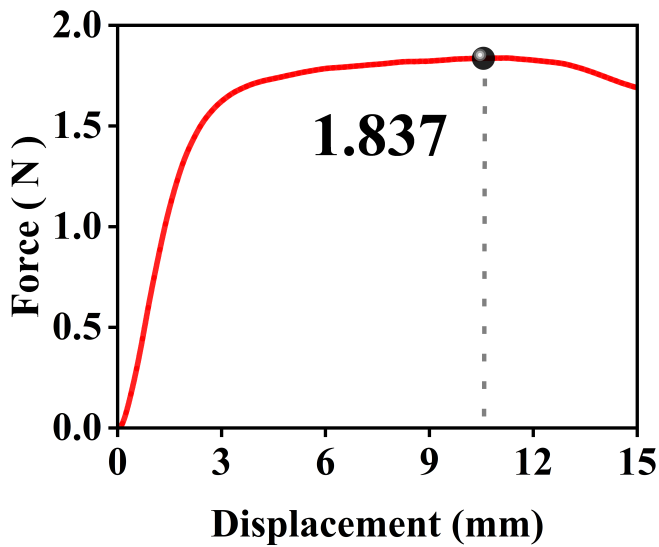
**

**Figure S18** Peel strength test curve of TPE@Mn@ZnS@AG@PVA.


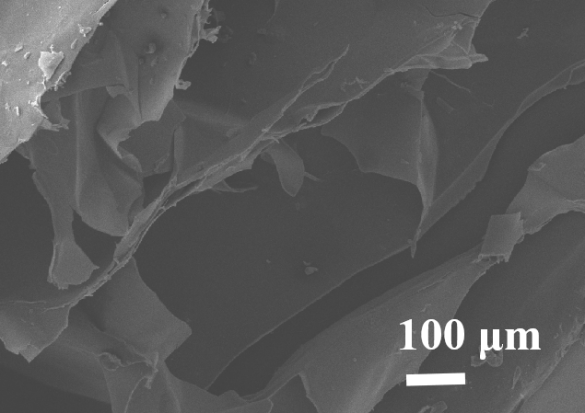


**Figure S19** SEM morphology of freeze-dried AG-PVA-borax hydrogel.


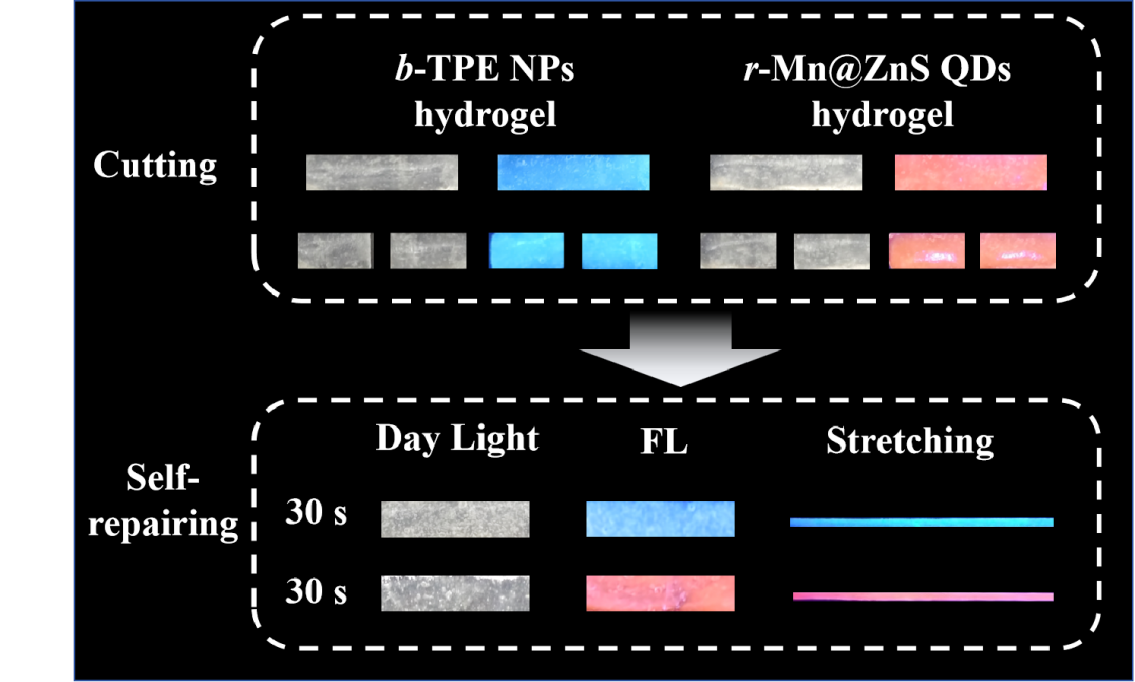


**Figure S20** Daylight and fluorescence images of cut (top) and self-repairing hydrogels (bottom).

**
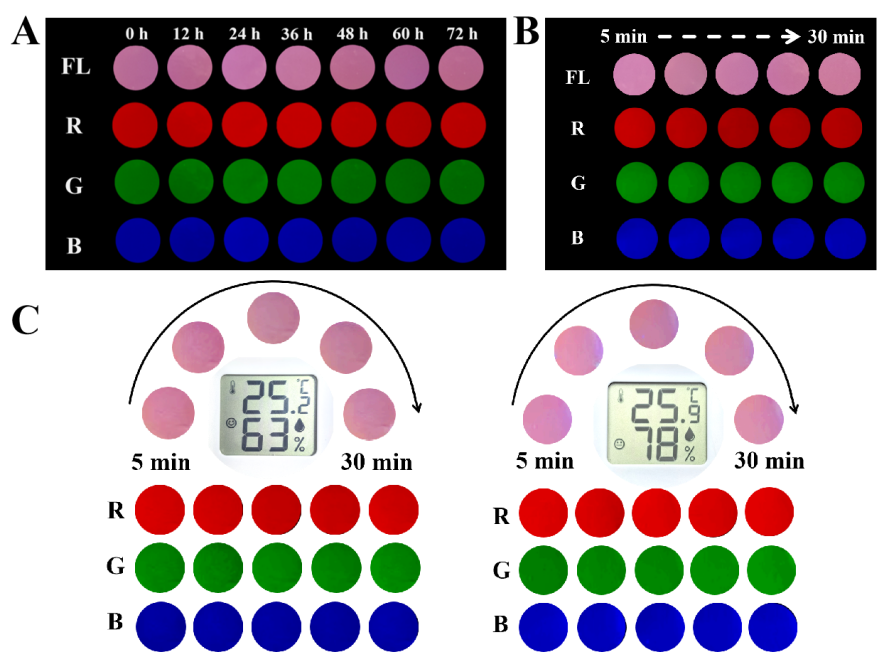
**

**Figure S21** Fluorescent color and RGB images of TPE@Mn@ZnS@AG@PVA after storing for different time (A), irradiating by 254 nm light for different time (B) and storing at different humidity for different time (C).

**
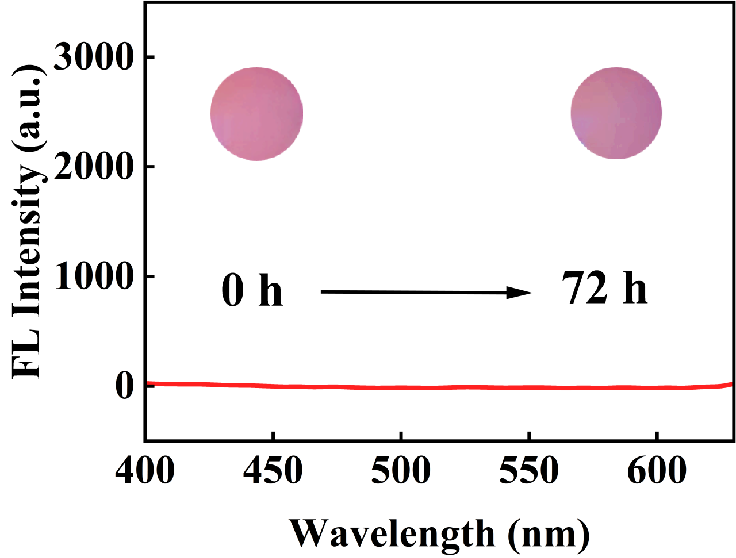
**

**Figure S22** Fluorescence spectrum of supernatant from TPE@Mn@ZnS@AG@PVA, and the fluorescent images after being immersed in solution for 72 h.

**
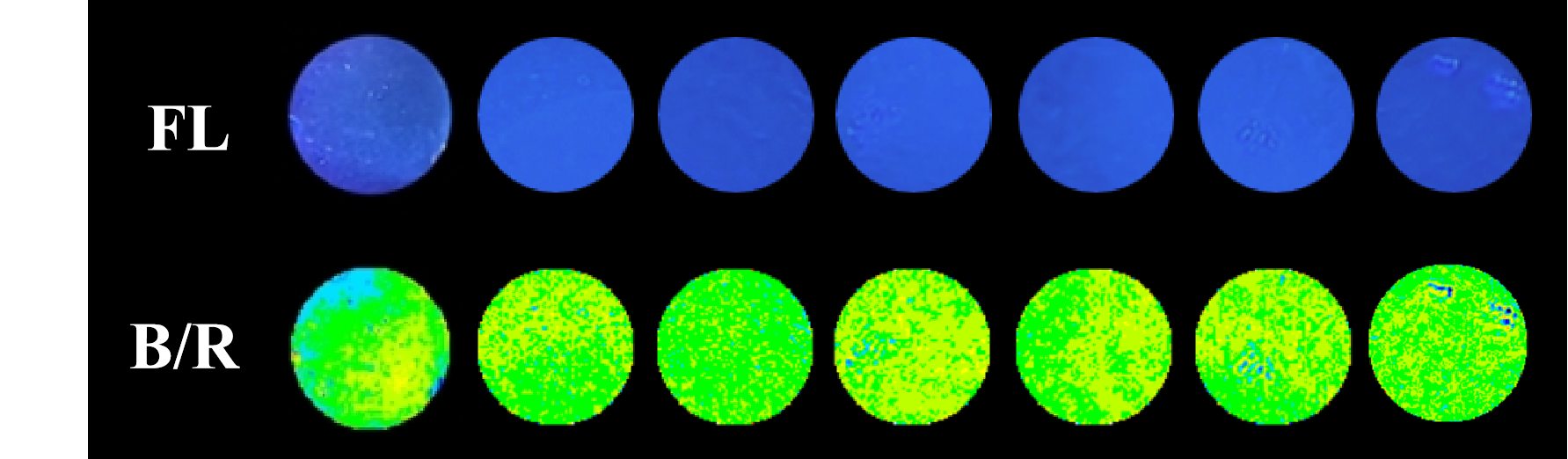
**

**Figure S23** Fluorescence photos and pseudo color of seven different prepared hydrogel patches being treated by 15 μg/mL TM.

**
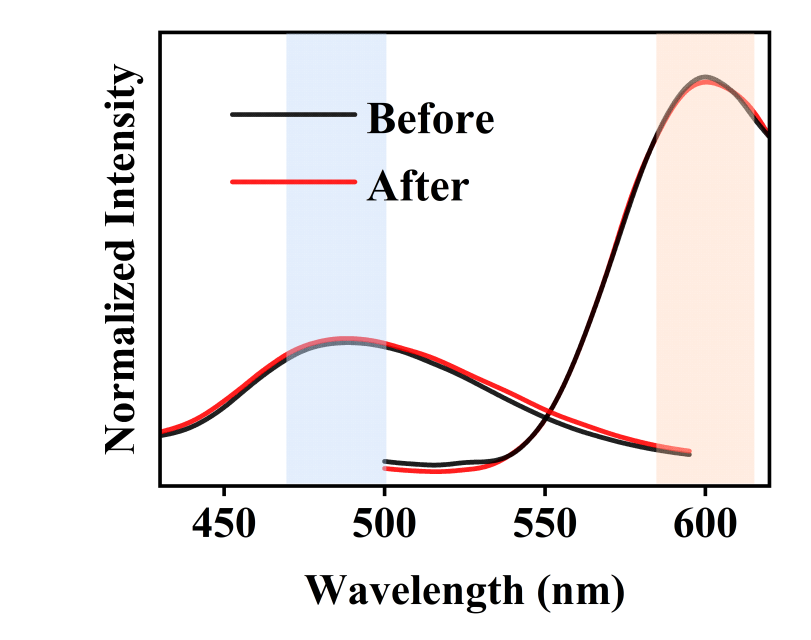
**

**Figure S24** Fluorescence spectra of *b*-TPE NPs (left) and *r*-Mn@ZnS QDs (right) before or after being stored on the surface of leaf of bok choi for 30 min.

**
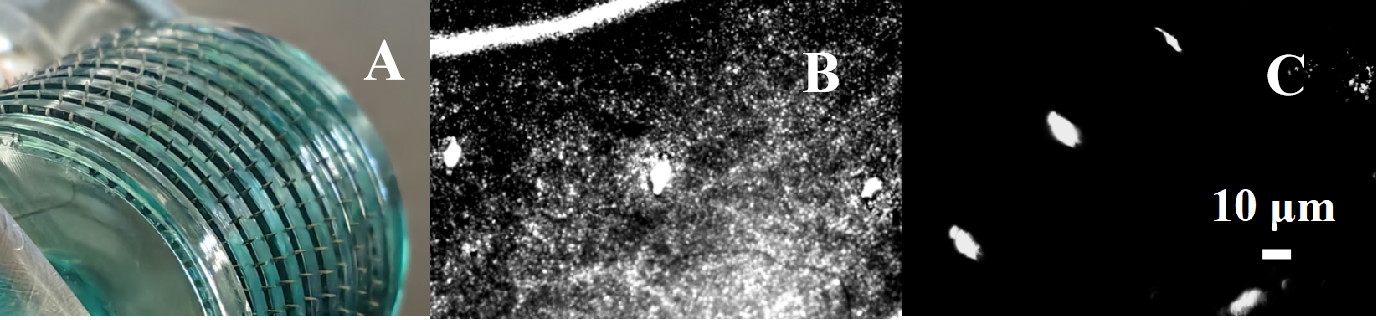
**

**Figure S25** (A) Photo of microneedle and (B-C) microscopic images of microneedle-treated leaf of bok choy.

**
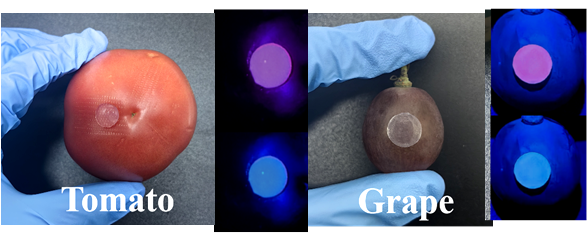
**

**Figure S26** Fluorescent color images of TPE@Mn@ZnS@AG@PVA being pasted onto the surface of tomato/grape sprayed without (up) and with (bottom) TM.

**
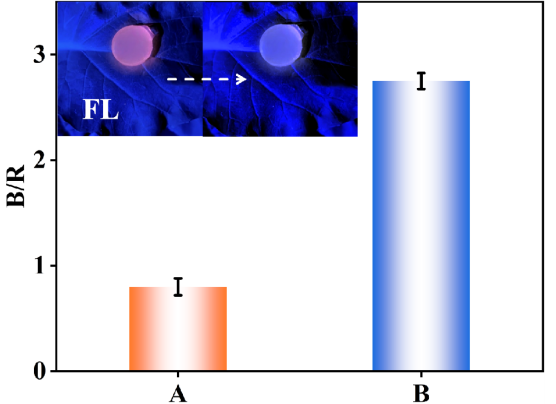
**

**Figure S27** Fluorescence color and B/R response of TPE@Mn@ZnS@Ag@PVA after being stored for 72 h toward bok choi sprayed without (left) and with (right) TM.

**
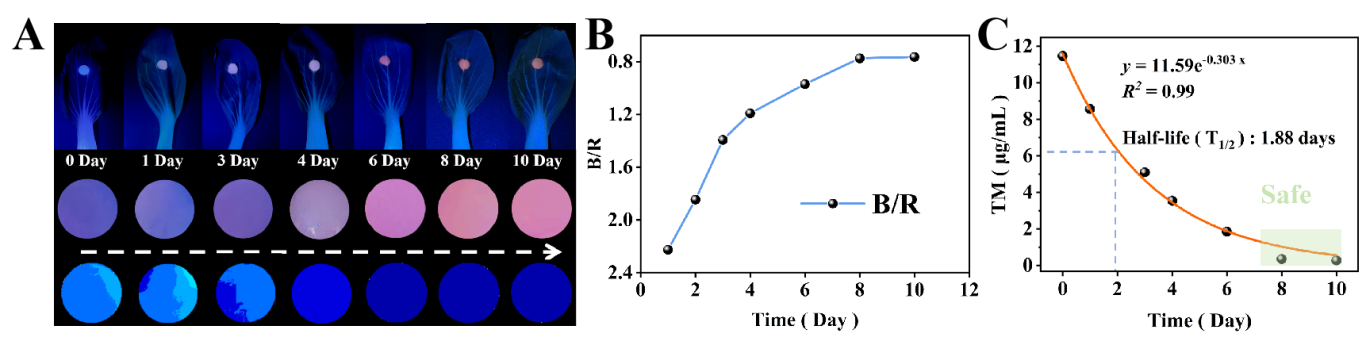
**

**Figure S28** Fluorescence and pseudo-color images (A), B/R (B) and TM concentration (E) of TPE@MN@ZnS@AG@PVA being pasted onto the surface of other batch of bok choy versus different interval days.

**References**

[1] Y. Yu, G. Huang, X. Luo, W. Lin, Y. Han, J. Huang, Z. Li, *Microchim Acta.* **2022**, 189, 325.

[2] Y. Han, X. He, W. Yang, X. Luo, Y. Yu, W. Tang, T. Yue, Z. Li, *Food Chem.* **2021**, 345, 128839.

[3] S. Tai, H. Cao, Y. Cui, C. Peng, J. Xu, Z. Wang, *Food Chem.* **2024**, 450, 139258.

[4] C. Cao, W. Guo, *Food Chem.* **2024**, 460, 140703.

[5] Y. Wang, Y. Ma, H. Wang, F. Shang, B. Yang, Y. Han, *Food Chem.* **2024**, 441, 138413.

[6] T. Wang, C. Xie, Q. You, X. Tian, X. Xu, *Food Chem.* **2023**, 424, 136479.

[7] Q. Shao, H. Lin, M. Shao, *ACS Omega* **2020**, 5, 10297.

[8] H. Zhu, N. Song, T. Lian, *J. Am. Chem. Soc.* **2010**, 132, 15038.
